# Physical Freezing in Children and Adolescents with Selective Mutism

**DOI:** 10.3390/bs16010152

**Published:** 2026-01-21

**Authors:** Shirley A. Landrock-White, Lindsay Lenton, Jean Victoria J. Roe, Chris A. Rogers

**Affiliations:** 1 Selective Mutism Information and Research Association, 5 Collingwood Drive, Sileby, Loughborough LE12 7NT, UKvroe@supanet.com (J.V.J.R.); 2 Bristol Medical School, University of Bristol, Bristol BS8 1NU, UK; chris.rogers@bristol.ac.uk

**Keywords:** selective mutism, autism, freeze response, children, survey, online questionnaire, child self-report

## Abstract

Selective mutism (SM) is an anxiety disorder that prevents speech in certain situations. Increasingly, it is reported that a proportion of those with SM may also be autistic and that physical freezing may be an important feature of SM. Information on speech and freezing behavior in children with a diagnosis of autism only (n = 20), SM only (n = 61), both autism and SM (n = 19), or neither diagnosis (n = 131) was collected via a self-selected cross-sectional online parent survey with an embedded child survey completed by a small subsection of the children (total n = 27: autism only n = 1, SM only n = 13, both autism and SM n = 3, neither diagnosis n = 10). Throat and body freezing were reported by children with SM, whether they were also autistic or not. The most common reasons given by the children that increased their difficulty in speaking were pressure to talk, worries about how they would be perceived, and fear of making mistakes. The Selective Mutism Questionnaire (SMQ) gave the lowest median score for children with both autism and SM, with median scores increasing in the order SM only, autism only, and neither diagnosis. Children who reported more freezing tended to have lower SMQ scores.

## 1. Introduction

Selective mutism (SM) is an anxiety disorder that usually commences in early childhood. Those with SM can speak fluently in some situations but remain consistently silent or unable to speak freely in others. The condition must last for at least one month and interfere with occupational or educational achievement and social interaction. It must not be better explained by another condition, lack of language knowledge, or occur only within the first month of attending a new setting (American Psychiatric Association, 2013; World Health Organization, 2022). SM is known to develop in many cases around the ages of 3 to 5 years, often when children first enter new settings and encounter new people (Driessen et al., 2020). With early intervention, the prognosis for recovery is good (Oerbeck et al., 2018), but in some cases, if left untreated, SM may persist right through a child’s school life and into adulthood. Children who are diagnosed with SM (n = 22), at age 7 or above, appear to be more impaired four or more years later (mean follow-up 4.2 years), with higher anxiety severity and lower adaptive functioning, than children diagnosed at a younger age (Kamani & Monga, 2020). They state, ‘When left untreated, evidence suggests that children with SM and SAD’ (social anxiety disorder) ‘may go on to have deficits in social skills, difficulties in academic functioning, psychiatric conditions in adulthood and higher rates of unemployment later in life.’

A review of research on SM by Kearney & Rede published in 2021 found evidence of wide heterogeneity and complexity in the behaviors and symptoms exhibited amongst people with SM (Kearney & Rede, 2021). Although the amount of research into SM is increasing, there is still insufficient knowledge about this complex condition to determine the etiology. The diagnostic manuals (Diagnostic and Statistical Manual (DSM) 5 and International Classification of Diseases (ICD) 11) list symptoms and not causes within the diagnostic criteria for SM (American Psychiatric Association, 2013; World Health Organization, 2022). SM is listed as an anxiety disorder in both manuals, and it is widely accepted now that anxiety is a significant feature of the condition. There are several hypotheses existing as to its cause and mechanism, varying in scope and complexity, with none fully explaining the wide range of behaviors some people with SM may display, in addition to an inability to speak in some select situations, whilst speaking fluently in others. These additional behaviors include anxiety (Dummit et al., 1997; Ludlow et al., 2023; Muris & Ollendick, 2021; Vogel et al., 2022a, 2024), sensory processing issues (Ludlow et al., 2023; Melfsen et al., 2021), communication disorders (Muris & Ollendick, 2021), developmental delay (Muris & Ollendick, 2021), avoidance of eye contact (Johnson & Wintgens, 2016), physical freezing, (Vogel et al., 2022a, 2024), problems using public bathrooms (Dummit et al., 1997; Johnson & Wintgens, 2016), and difficulties eating in the company of other people (Dummit et al., 1997; Ludlow et al., 2023; Melfsen et al., 2021; Muris & Ollendick, 2021; Vogel et al., 2022a, 2024).

Some authors have questioned whether anxiety is an etiological factor in SM or a symptom of it. A meta-analysis of 22 studies (N = 837) found that 80% of children with SM also had another anxiety disorder, with 69% being SAD. The authors acknowledged, however, that this did not answer the question as to whether SM was an anxiety disorder itself or just a symptom of anxiety (Driessen et al., 2020). A smaller study in 54 children with SM found that 74% of the children had a comorbid anxiety disorder and that parents of these children were significantly more likely to have had a history of excessive shyness or social anxiety than parents of children without SM, suggesting familial transmission of SM and SAD (Kristensen & Torgersen, 2001). Some authors question whether SM is a type of SAD (Scott & Beidel, 2011), whilst others argue that they are distinct, but closely related disorders (Gensthaler et al., 2016).

Behavioral inhibition (BI), an early childhood temperamental disposition characterized by sensitivity to novel experiences leading to fear and avoidance of unfamiliar places or people, has also been implicated as a precursor of SM; however, it is unclear as to whether this is related to SM or comorbid SAD, since BI is also known to be a precursor to SAD. Gensthaler and colleagues found that infant BI was higher in those with lifetime SM than those with SAD; however, Muris et al. found that an apparent relationship between BI and SM was no longer significant when compensating for SAD symptoms, suggesting a possible indirect relationship between BI and SM mediated by SAD (Gensthaler et al., 2016; Muris et al., 2016).

In the past ten years, there has been an increase in the anecdotally reported incidence of young people who have SM and are also autistic, but there has been little research into this subject. The diagnostic criteria for autism are, in summary, persistent deficits in social communication and social interaction, persistent restricted and repetitive behaviors, interests, or activities, symptoms are present during early development, and symptoms/characteristics cause significant impairment in social, occupational, or other important areas (World Health Organization, 2022). Poor eye contact, mutism, anxiety, hyper-sensitivities, and intolerance of minor changes in routine are all symptoms that can occur in both SM and autism, giving the potential for misdiagnosis or failure to assign co-occurring diagnoses where appropriate (Johnson & Wintgens, 2016).

In a Swedish study of 97 children with a diagnosis of SM, Steffenburg and colleagues found 63% of their study group to also be autistic (Steffenburg et al., 2018). A literature review on the prevalence of anxiety disorders within autistic individuals revealed a reported prevalence range of between 42% and 79% (Kent & Simonoff, 2017). The causes of anxiety within autism are not as yet well understood but are believed to be complex with anxiety being caused by a combination of multiple factors, such as sensory sensitivities, differences in brain morphology in some autistic people, which may be a distinct co-occurring condition, differences in neurological responses to stressors, difficulty recognizing and understanding emotions, and intolerance of uncertainty (Rodgers & Ofield, 2018).

Two studies in the Netherlands in children aged 3 to 6 (N = 172) and 6 to 12 (N = 169) found positive correlations between SM and SAD, autism, and BI (Muris et al., 2021, 2024), and Koskela and colleagues found a wide range of conditions amongst siblings of children with SM, with the closest association being with emotional disorders and autism (Koskela et al., 2024).

### Physical Aspects of SM

Information and research into SM typically focus on the psychological aspects of the condition. Physical difficulties have been described in only a few studies (Schwenck et al., 2022). People with SM often say that their throats feel blocked or tight when expected to speak, and some describe feeling frozen, with muscles in their bodies becoming stiff, limiting their movement. The extent to which SM may involve physical freezing is unknown.

It has been suggested that a freeze response is expressed in immobility, including motor and vocal inhibition, which are typical symptoms in children with SM (Schwenck et al., 2022). In a study (n = 86) where parents of children with SM were asked to describe their children’s symptoms, other than failure to speak in situations where speech was expected, 65% described freezing-like behavior, such as immobility of the whole or parts of the body, increased body tension, slowed motor function, or reduced responsiveness. Fear was cited by 66% of the parents (Vogel et al., 2024). These symptoms potentially fit with the freezing or tonic immobility stages of the Defense Cascade theory (which also includes the fight or flight response) when a person is exposed to a perceived threat (Kozlowska et al., 2015). Freezing is thought to act as a parasympathetic brake on motor response when under threat in preparation for the active parts of the fight or flight response. During freezing, the heart rate is lowered, and it then rises again in the active stages. Flexibility of transition from one stage to another is highly important in coping with stress (Roelofs, 2017).

A study (n = 96) in which children with SM were given verbal and non-verbal tasks while heart rate, respiration, and skin conductivity were measured found that children with SM who did not speak during the verbal task had a lower heart rate and lower skin conductivity compared to children with SM who managed to speak. These findings, the authors suggested, could be explained by a reduction in anxiety as a consequence of not speaking because of maladaptive emotion regulation or through the use of an avoidance mechanism in social situations where the child is expected to speak. Alternatively, a reduction in heart rate could be due to the children experiencing a physiological freeze response (Vogel & Schwenck, 2021).

Fox and colleagues observed that BI preschool children, ‘when challenged in a novel situation’, were more likely to freeze than non-BI peers and were less flexible in social problem solving. They hypothesized that there are two control processes that moderate outcomes for the children: automatic control, which they defined as immediate, reactive changes in attention or sensorimotor inhibition that are in direct response to a stimulus or event, and planful control, which they defined as prolonged and proactive changes in attention or sensorimotor inhibition in support of a specific goal. They suggested that BI predicts lower levels of planful control and higher levels of automatic control. The reasons for this are unclear but may be related to early social experiences (Fox et al., 2021).

In their ‘unsafe world’ model, Melfsen et al. postulated that SM is caused by high sensory processing sensitivity, which leads to freezing or dissociative symptoms because of ‘an automatic stress reaction in situations erroneously classified via cognition without awareness as ‘unsafe’’ (Melfsen et al., 2021). They measured sensory processing sensitivity and levels of dissociation using standardized scales in populations of SM and non-SM children and adolescents and found that sensory-processing sensitivity was significantly higher in the group of children and adolescents with SM. Levels of dissociative symptoms were also significantly higher in the SM group but did not exceed clinical threshold levels for dissociative disorders (DDs). They concluded that anxiety in SM may develop because of repeated stress experiences causing dissociation or freezing, rather than anxiety being the root cause, and that this would have important implications for treatment, which currently usually uses cognitive behavioral methods and/or small steps graded exposure. They suggest that the use of sensory processing therapy to improve regulation of the nervous system, with the aim of dampening the automatic stress response, could be an important part of any treatment for SM.

Sensory hyperactivity has also been found to occur within autism. Tavassoli et al. found a significant positive correlation between autistic traits in adults and sensory over-responsivity, and Taylor et al. found that autistic females had significantly greater levels of sensory hyperactivity than non-autistic women (Tavassoli et al., 2014; Taylor et al., 2020).

Catatonia has been widely reported in autism and neurodevelopmental disorders (NDD), with meta-analysis showing a prevalence of catatonia of 10.4% within the autistic population (Vaquerizo-Serrano et al., 2021) and an estimated prevalence within NDDs of between 6 and 20.2% (Moore et al., 2022). Catatonia, as defined within the DSM-5 and ICD11, gives several symptoms similar to those of many people with SM when they are placed in a situation where speech or communication is expected and they seemingly become ‘frozen’ in place, such as decreased, increased, or abnormal psychomotor activity, staring with a fixed gaze, withdrawal of interaction with others, immobility, mutism, or very little verbal response or whispering to the point of being unintelligible and varying degrees of rigidity of muscle tone (American Psychiatric Association, 2013).

Moskowitz suggested that catatonia could be better understood as a fear response similar to tonic immobility, whilst Shah and Wing argued that stress was frequently a factor preceding the deterioration of catatonic-like states in autistic people (Moskowitz, 2004; Shah & Wing, 2006).

Debate is ongoing as to whether catatonia within autism is true catatonia by DSM-5 criteria or could be explained more as freezing behavior (Moore et al., 2022).

The aims of this study were to assess the frequency of SM amongst children and young people with and without a co-diagnosis of autism in the study population and to investigate differences in freeze response in physically preventing speech as reported by the children and young people. A secondary exploratory aim was to assess whether there are differences between biological boys and girls with respect to freezing. The study does not itself test directly the mechanistic models of freezing described above, which instead serve as a conceptual backdrop, but gathers information directly from children themselves on their freezing experiences.

The hypotheses for this study are the following. 1. Children and young people with SM only or both autism and SM diagnoses would have lower Selective Mutism Questionnaire (SMQ) scores compared to those with autism only or neither diagnosis. 2. The children and young people with both autism and SM would experience the greatest life interference related to talking, followed in decreasing magnitude by those with SM only, autism only, and neither diagnosis. 3. The children and young people with neither diagnosis would experience the least throat freezing, followed in increasing frequency by those with autism only, SM only, and both autism and SM.

## 2. Materials and Methods

### 2.1. Parent Survey

Between February and September 2023, social media snowballing was used to advertise a parent survey to capture data on the speaking behaviors of children between the ages of 5 and 16 with clinical diagnoses of SM, autism, both, or neither (neurotypical/typically developing children). Adverts, written in English, inviting participation from parents/carers (hereafter referred to as ‘parents’) with children with or without a clinical diagnosis were placed on the Selective Mutism Information and Research Association (SMiRA) website, www.selectivemutism.org.uk, and on SMiRA Facebook groups (>23,000 members) covering the wider SM community, including those with SM, parents, professionals, and researchers. The survey was also advertised on the UK National Association for Special Educational Needs (NASEN) site, the UK SM Clinical Excellence Network (CEN) Facebook group, and personal Facebook pages with a request that those who read the advert share it more widely with their own social media communities.

Participants were asked to follow a web link to an anonymous online survey where they were presented with information and instructions on the initial screen. After indicating their informed consent, participants responded to demographic questions before the main body of the study commenced. Respondents were next asked to indicate for each of their children individually if their child had a diagnosis of autism, SM, neither, or both. They were then presented with the SMQ for each child reported (Bergman, 2012). This measure consists of 17 items of speaking behavior divided into three sub-domains, family, school, and social situations, to which parents indicate how frequently, within the last two weeks, the statement has been true for their child using a four-point Likert scale (never, seldom, often, always). This section of the questionnaire is used widely for diagnostic purposes and has been shown to have good reliability (Oerbeck et al., 2020). The SMQ concludes with an additional six questions used for clinical purposes only (not for diagnosis), to give an indication of how much interference/distress is experienced by the child due to not speaking. This last section uses a four-point Likert scale (not at all, slightly, moderately, extremely).

Upon completion of all questions in the parent survey, participants were asked whether they would be willing to allow their children to fill in a shorter online questionnaire, with supervision and support from the parent if needed.

### 2.2. Child Surveys

Parents who consented to allowing their children to take part in the child survey were emailed links to online Qualtrics questionnaires for 5–10- and 11–16-year-olds. The questionnaires were designed to take around 15 minutes to complete, depending on the length of the answers given by the children. No incentives were offered, but children in the 5–10 years group were presented with a cartoon at the end to watch as a reward. Parents were asked to sit with children under the age of eleven to assist them if necessary and to ensure that they were not upset by completing the questionnaire. Before starting the questionnaire, parents were asked to share an introductory information sheet with 5- to 10-year-old children to ensure they understood what they were being asked to do and that they consented to do it. It was emphasized that parents should not put any pressure on the child to complete the survey and that they could stop at any time. The children and young people aged 11 to 16 years were presented with an introduction explaining the survey and were then asked to complete a tick box consent form before continuing to the questions. They were also told that they could stop at any time. Parents were asked to offer support to the older age group but to allow them to complete the questionnaire independently if they preferred.

The two questionnaires contained the same questions, but with age-appropriate language being used in each case. Questions asked about the experience of physical freezing symptoms, where in the body it was felt, if at all, and how this related to speaking behavior. See the Supplementary Materials for copies of the questionnaires used.

### 2.3. Statistical Analysis

Category data are presented as numbers and percentages; scores are summarized using the mean and standard deviation (SD) or median and interquartile range (IQR) if the distribution is skewed. SMQ scores were derived for the three sub-domains, along with the total of the three. Scores for the family and school domains range from 0 to 18, and the social domain ranges from 0 to 15. Scores are divided by the total number of questions contributing to the score so that all scores are presented in the range 0 to 3, with lower scores indicating that interactions/conversations are more difficult. The same approach was used to score the interference question responses, namely, the four options were scored from 0 (never) to 3 (always), and the sum of the scores for the six questions was divided by six to give a final inference score in the range 0 to 3. For interference, higher scores indicate greater interference. Scoring the interference questions was exploratory, and the score derived has not been validated.

Children who were home schooled (n = 17) are excluded from all SMQ analyses, as the school-related questions were not completed for these children. The SMQ, when completed, was completed in full, and no allowance for partially missing data was needed when deriving the scores. Internal consistency of responses was assessed using Cronbach’s α statistic. The Kruskal–Wallis test was used to compare the scores across the diagnostic groups, and Spearman’s rank correlation was used to assess the association between the impact and interference scores. There was no adjustment of *p*-values for the number of statistical comparisons made, and no post hoc analyses were undertaken. Analyses were performed using Stata, version 18.0 (StataCorp, College Station, TX, USA).

## 3. Results

### 3.1. Parent Survey

**Figure 1:**
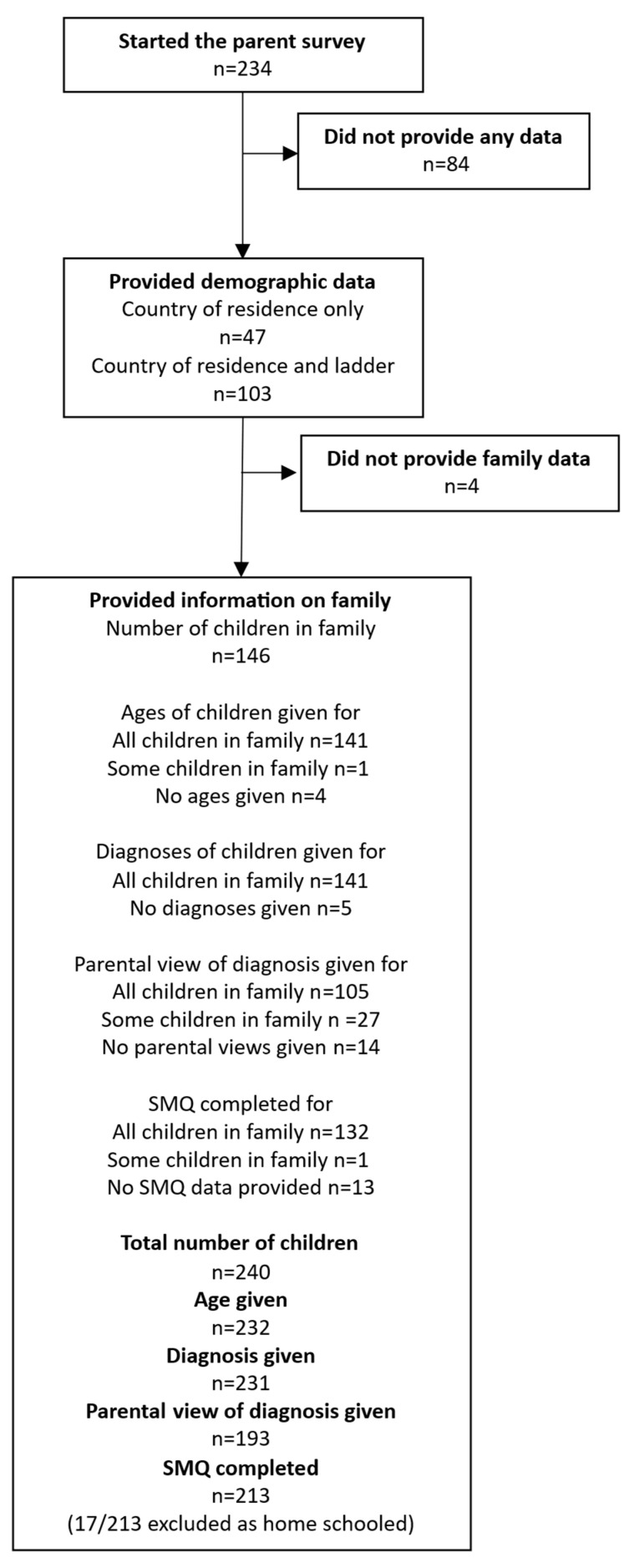
Summary of data obtained from the parent survey. The ladder refers to the demographic ladder representing the position the respondent felt they occupied in society.

In total, there were 234 parent responses to the survey. Overall, 77/234 surveys were marked as completed (33%), and a further 51/234 (22%) were over 95% complete. Almost 40% of surveys had less than 15% completion (91/234, 69 with 1% completed). These people answered some or all of the introductory questions but did not answer the questions about their children. Overall, 103/234 respondents (44%) completed the demographic ladder, where a score of 10 indicated people with more money, more education, and jobs with more recognition, and 0 indicated people with less money, less education, and jobs with less recognition. Responses ranged from 0 to 10, with a mean response of 5.5 (SD 1.8). Country of residence was completed by 150/234 (64%) respondents, most of whom were based in the UK (Table 1). Most parents who provided data on their country of residence went on to provide data on their children (Figure 1).

### 3.2. Number and Ages of Children in the Family

**Figure 2:**
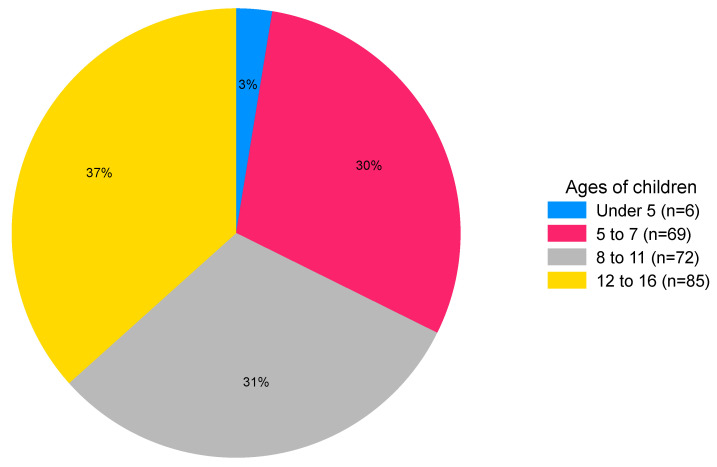
Chart showing the percentage of children in each age band (n = 232).

This question was completed by 146/234 (62%) respondents. Over 85% of respondents had one or two children (Table 2). No data were obtained for the one family with five or more children. Of the 240 children, 232 were aged between 3 and 16 years (median 10 years, IQR, 7–13 years). There were similar proportions of children of infant (ages 5 to 7 years), primary (ages 8 to 11) and secondary school age (ages 12 to 16) (Figure 2).

### 3.3. Diagnoses of Autism and SM in Children

A diagnosis was given for 231/232 children, with the age reported. There were 39 children with an autism diagnosis (17%) and 80 with a diagnosis of SM (37%); 19 of these children had both diagnoses, and 131 (57%) children had neither diagnosis (Table 3).

Parental view of the child’s diagnosis was given for 193/231 (84%) children. The question was answered for 17/20 children with a diagnosis of autism only. Twelve parents felt they had autism, two felt they had SM, and two felt they had both. One respondent said they felt the child had neither SM nor autism despite the diagnosis. The question was answered for 45/61 children with a diagnosis of SM only; thirty-one parents agreed with this diagnosis, twelve felt they had autism as well, one reported autism only, and one said neither despite the diagnosis. The question was not answered for the 19 children with both diagnoses. Table 4 provides an overview of the agreement between the child’s clinical diagnosis and the parental view. In 64/231 (28%) of cases, the parent felt the child was underdiagnosed (lighter shading in Table 4), and in 5/231 (2%) instances, they felt the child had an incorrect diagnosis (darker shading in Table 4).

### 3.4. SMQ Scores—Impact on Family, School, and Social Situations

**Figure 3:**
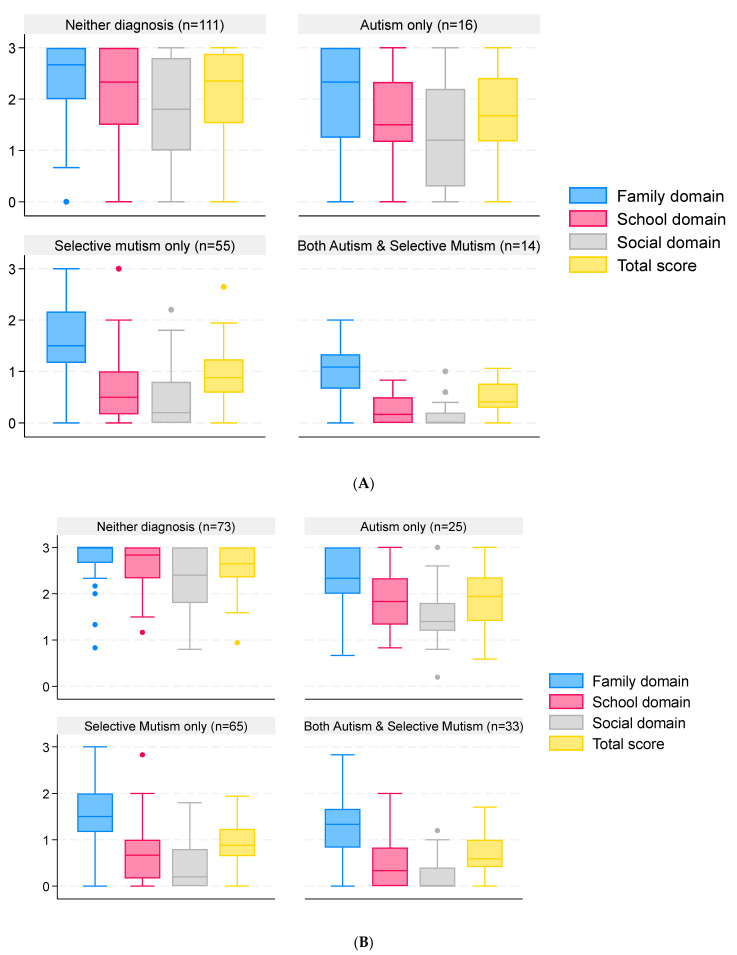
SMQ scores of children and young people grouped by reported clinical diagnosis and parental view of the diagnosis. Data are median and interquartile range (box) for SMQ scores in each domain and in total. Whiskers are lowest observed value above [lower quartile − 1.5 × interquartile range] and highest observed value below [upper quartile + 1.5 × interquartile range]. Dots represent observations outside the range covered by the whiskers. Scores range from 0 to 3, with lower scores indicating greater impact. Responses are grouped by clinical diagnosis (**A**) and by parental view of the child’s diagnosis (**B**).

Excluding the home schooled children (n = 17), data on the SMQ were provided for 196 children from 133 families. Overall, the reliability of the responses was excellent (Cronbach’s α of the three subscales was 0.93, and when including the total score, it was 0.96). Scores for each domain by diagnostic group are illustrated in Figure 3, and summary scores are given in the Supplementary Materials. The total scores varied by diagnostic group (*p* < 0.0001), with lower SMQ scores for children with SM and both autism and SM (Figure 3A). When grouping children by the parents’ view of their diagnosis, a similar pattern was observed (Figure 3B).

### 3.5. SMQ Scores—Interference of Not Talking on Family, School, and Social Situations

**Figure 4:**
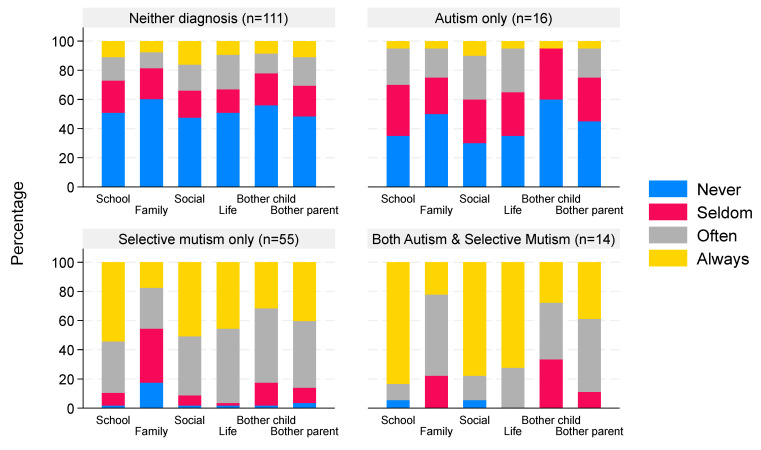
Interference of not talking grouped by reported clinical diagnosis. Data are the percentage of children with a degree of interference that not talking has on different aspects of life. The percentages within each bar sum to 100%; the *x*-axis categories correspond to family, school, social domains, and perceived bother for child/parent.

**Figure 5:**
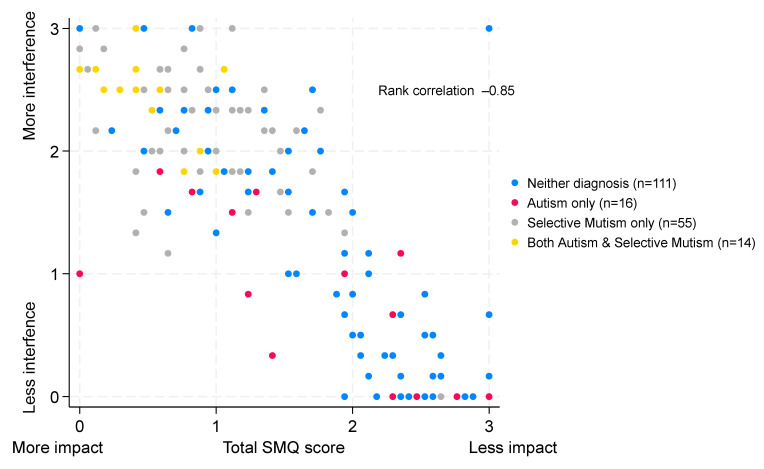
Correlation between the total SMQ score and the interference of not talking. Scatter plot of SMQ scores and interference of not talking scores. Both scores range from 0 to 3.

The median score for children with neither diagnosis was 0.33 (IQR 0–1.67, n = 111) compared to a median of 0.92 (IQR 0 to 1.5, n = 16) for autism-only children, 2.17 (IQR 1.83–2.5, n = 55) for children with a diagnosis of SM only, and 2.5 (IQR 2.33–2.67, n = 14) for children with both autism and SM, indicating greater interference of not talking amongst children with a diagnosis of SM (*p* < 0.0001 comparing ‘scores’ across the four groups). The extent of interference of not talking by diagnostic group is illustrated in Figure 4, and summary scores for each diagnostic group are given in the Supplementary Materials. There was a strong negative correlation (ρ = −0.85, 95% CI −0.89 to −0.80) between the total SMQ score and the interference ‘score’, as illustrated in Figure 5.

### 3.6. Child Survey

**Figure 6:**
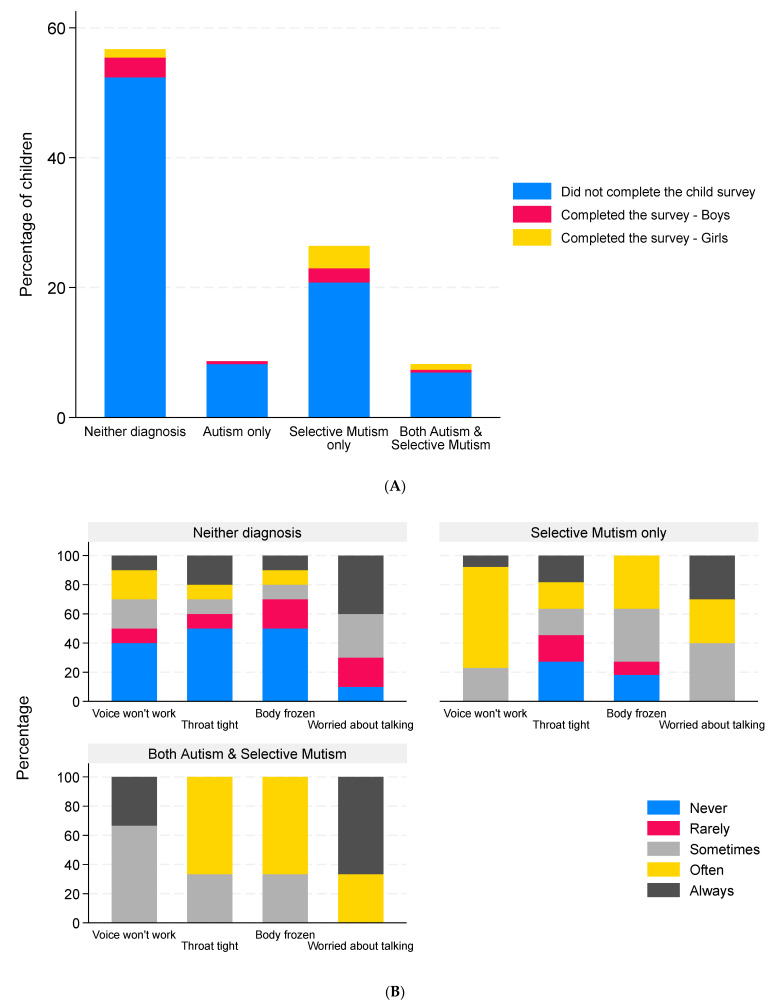

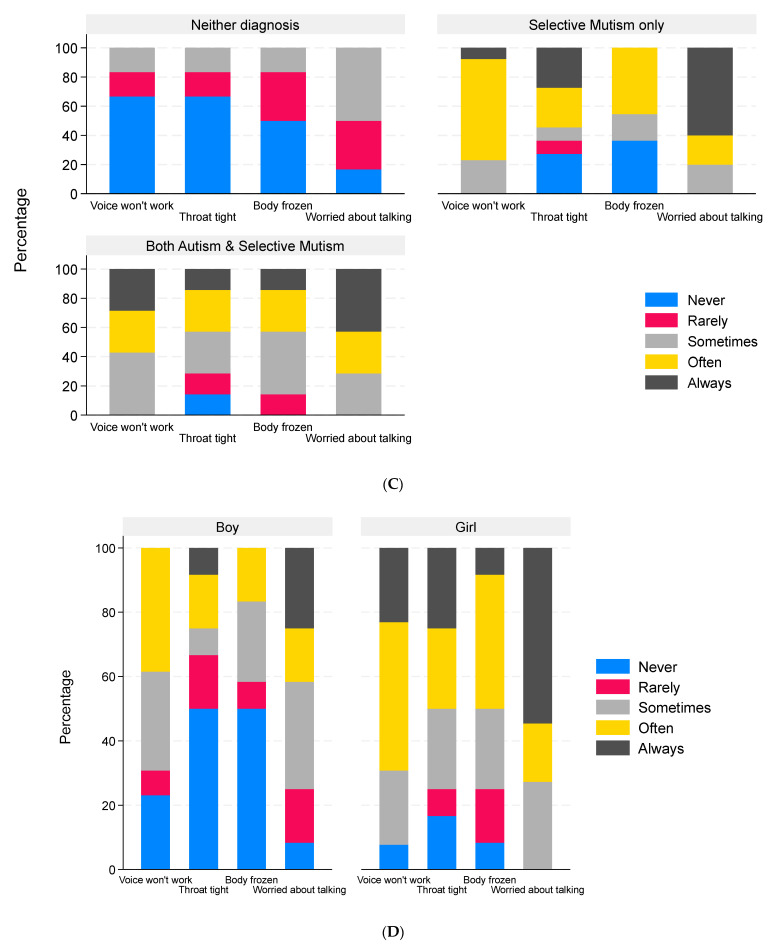
Children’s responses to the child survey. Data are the percentage of children in each diagnosis group who did or did not complete the child survey stratified by sex ((**A**), n = 231, the three child surveys that could not be matched to the corresponding parent surveys were omitted, and these are included in the non-responder group); percentage of children reporting different levels of freezing of the voice (n = 26), throat (n = 24), and body (n = 24) and the degree to which they were worried about talking (n = 23) with responses grouped by reported clinical diagnosis (**B**), by parental view of the child’s diagnosis (**C**), and by biological sex of the child (**D**).

Twenty-two children aged between 5 and 10 years and eight children aged between 11 and 16 years consented and responded to the child survey. It was not possible to determine the diagnosis for three children in the 5–10 age group, as they could not be matched with the corresponding parent survey. These responses were omitted from the analysis. Of the remaining 27 children from 25 families, 14 responses were from boys and 13 were from girls. The median age of the children was 8 years (IQR 5–11, range 5–16). The four respondents over the age of 12 were all girls. Ten children (37%) had neither diagnosis, one had autism only, thirteen had SM only (48%), and three had both autism and SM (11%). The single autism-only child did not provide any data other than their sex, age, and how many languages they spoke. Proportionally more children with SM responded to the survey compared to the other diagnostic groups (Figure 6A). The SMQ scores of the survey responders were similar to the scores for the children and young people who did not complete the survey (Table 5). A similar proportion of parents of these 27 children disagreed with the child’s diagnosis (26%), as was seen for the parent survey (30%). Four of the twenty-seven children (15%) spoke a second language; these children had all been diagnosed with SM only.

### 3.7. Freezing Behavior Reported by the Children and Young People

The freezing behavior reported by the children and young people is illustrated in Figure 6B–D. The child with autism only did not provide data on freezing behavior. Children with SM only or both autism and SM experienced freezing of the voice, throat, and body more frequently than the children with neither diagnosis (Figure 6B). The children with neither diagnosis who experienced freezing on a regular basis were all in the group whose parents felt they should have a diagnosis (Figure 6C). In terms of body parts affected, the median number of parts affected was 1.5 (IQR 0–3, range 0–6). Different body parts were affected with similar frequency (Table 6). Most children (20/23, 87%) were worried about talking at least some of the time.

In terms of the child’s feelings when expected to speak, responses were provided from eleven children (one of infant school age, six of primary school age, and four of secondary school age). Most frequently reported feelings were experiencing pain/discomfort/funny feelings in the tummy (n = 3), feeling scared (n = 3), and feeling sick (n = 2). Other feelings described included knocking inside the head leading to crying, voices in the head discouraging speech and movement, ‘bogey’ in throat, sweating, racing heartbeat, and fidgeting. Most of the children (10/12, 83%) who experienced discomfort when expected to speak either had a diagnosis of SM or their parents suspected they did. Both children and parents in this group were concerned at times about their speaking behavior. The children with neither diagnosis mostly did not express fear of talking, and their parents were much less concerned about their speaking behavior. Sixteen children gave details of situations or actions that increased their anxiety about speaking; five children did not identify any specific actions or situations (four of the five had SM or suspected SM), and the remainder (n = 7) left the question blank. Of the twenty responses provided, seven were from children of infant school age, seven were from children of junior school age, and six were from children of secondary school age. Actions and situations that increased the child’s anxiety around speaking included feeling pressure to speak, including impatience on the part of the person asking the question (e.g., repeating the question whilst waiting for an answer, being told to hurry up), what the audience/person will think of their answer (e.g., fear of getting the answer wrong and appearing silly, fear of misunderstanding the question), and how it is said (e.g., croaky or strange voice, incomplete sentences due to throat freezing), also caused greater worry, as did being asked multiple questions. The size and composition of the audience, for example, if the questions were being asked in a one-to-one setting or a bigger group and if the person asking the question was known to the child or young person or not, were other factors. Loud speaking and a feeling of being stared at were also mentioned.

### 3.8. Responses from Boys and Girls

The boys who completed the survey were, on average, younger and had a higher proportion without a diagnosis of SM or autism than the girls. Differences in SMQ scores, severity of symptoms, and body parts affected between the boys and girls reflect the diagnoses of the children (Table 6 and Figure 6D). In terms of the child’s feelings when expected to speak, eight of the eleven responses were girls, but equal numbers of boys and girls answered the question about what actions and/or situations made them more worried when they were expected to speak.

## 4. Discussion

Most responses to the parent questionnaire were from UK residents with one or two children aged between 5 and 16 years. There was a good balance of children across the school years: infant (age 5 to 7), junior (age 8 to 11), and secondary years (age 12 to 16). The data suggest that children obtain diagnoses for autism after the age of 7 years, whilst SM is mostly diagnosed between the ages of 5 and 7 years. This difference could be because some children recover and no longer meet diagnostic criteria for SM as they get older, or because failure to speak is more easily noticed than neurodiversity. The peak age for a diagnosis of both autism and SM was between 11 and 16 years, with relatively few children receiving both diagnoses at an earlier age. It is possible that the presence of SM is delaying or hindering assessment for autism. This observation is based on our self-selected sample; a purpose-designed study with longitudinal diagnostic data is needed to examine this further.

### 4.1. The Proportion of Children with SM Who Are Also Autistic

Nearly a quarter (24%, 95% confidence interval 15–35%) of the children with SM in this study were also autistic, with parents suspecting that this figure may be even greater; however, it should not be taken as an estimate of population prevalence due to the self-selected nature of the sample recruited via SM social media networks. Whilst the proportion of children with a diagnosis of both autism and SM in our study is not as high as the 63% reported by Steffenburg et al., it still indicates the importance of considering whether a person presenting with SM may also be autistic during diagnosis and therapy to understand fully their needs and tailor treatment (Steffenburg et al., 2018). Shimoni and colleagues suggested that SM may be an ‘early marker’ for autism based on a small sample of girls diagnosed with SM at the age of three or four, who were some years later also diagnosed with autism (Shimoni et al., 2025). Misdiagnosis of autism in children with SM is also possible, given the difficulties in assessing a child for autism who cannot speak during assessments. A large overlap of apparent symptoms and behaviors, and co-existing conditions such as SAD, BI, and sensory processing difficulties are seen in children with SM and autism. Snyder et al. urged caution in diagnosing young children with autism who presented with SM-like behaviors based on limited observations (Snyder et al., 2008). It is known that, even in the absence of SM, assessment for autism can lead to a significant level of misdiagnosis (false positive or false negative diagnoses). Greene and colleagues found 34% false positive and 1% false negative indications for children aged five to sixteen when using the Autism Diagnostic Observation Schedule 2 (ADOS2), particularly in children with high levels of anxiety, emphasizing the need for multimodal assessment (Greene et al., 2022). A study on adults who had been given diagnoses other than autism prior to later being diagnosed as autistic revealed that a quarter of them believed they had previously been misdiagnosed, and 6.6% had anxiety disorders (Kentrou et al., 2024). Hus & Segal, in a review of the challenges surrounding the diagnosis of autism in children, recommend, to avoid misdiagnosis, the use of early diagnostic tools, long-term surveillance to assess diagnosis stability and severity, individual ‘Functional Communication Profiles’, the development of autism diagnostic tools more applicable to girls, and the consideration of possible comorbidities. When discussing autism and SM, they state, ‘The need for increased vigilance in differential diagnosis in disorders with overlapping symptoms cannot be over-stated.’ (Hus & Segal, 2021).

Seventy percent of parents agreed with the clinical diagnoses given to their children. Where they disagreed, most parents (85%) felt that they should have additional diagnoses; 4% of the children were suspected by their parents of having both autism and SM, but they did not currently have a dual diagnosis. Whilst parental perceptions of under- or overdiagnosis may be influenced by their knowledge of autism and SM, and by their own mental health, if the parental suspicions are correct, then that would increase the percentage of children with SM in the study who are also autistic to around 28%. This may still be an underestimate due to difficulties encountered in obtaining diagnoses of both autism and SM (Keville et al., 2024). Petrou and colleagues found that girls were diagnosed with autism on average one year later than boys, and that underdiagnosis was common (Petrou et al., 2018). There is still a lack of awareness of SM in schools and health settings, which may delay diagnosis (Adamson et al., 2025; Douglas, 2021). While the DSM has recognized dual diagnosis since 2013, the ICD manual still lists autism as an exclusion for SM, but it also states that in autism, unlike SM, ‘language and communication impairments are notable across environments and social situations’, which suggests that dual diagnosis is possible (American Psychiatric Association, 2013).

### 4.2. Differences in Speaking Behaviors

Children with a diagnosis of SM only had a significantly lower average total SMQ score than those who had autism only (*p* = 0.001) and a higher average total score than those who had diagnoses of both autism and SM (*p* = 0.004). These results are similar to those of Ludlow et al., who also found significantly lower total scores for children with both autism and SM when compared to those with SM only (Ludlow et al., 2023). If compared at the domain level, our results are also similar to theirs for the family (entitled home in their study) and social domains, where children with both autism and SM had lower scores than children with SM only. The results differ in the school domain, where they found that autistic children with SM had significantly higher scores than those with SM only. In contrast, the pattern was reversed in our study; on average, those with SM only had higher scores than the both autism and SM group. Reasons for the differences between the school domains in the two studies may possibly be explained by differences in the ages of the children. In our study, the average age of children in the SM-only group was 8.4 years (n = 61), and in the both autism and SM group, it was 12.4 years (n = 19) compared with 10.5 years (n = 37) and 9.0 years (n = 38), respectively, for the Ludlow study. Extra stresses and difficulties in coping within a secondary school setting compared to infant and junior school may have increased the anxiety of autistic children with SM more than for children with SM alone, with a greater impact on their speaking behavior. Speaking behavior is reduced in children with SM only, even in the family domain, compared to children with neither diagnosis and autism only, but the differences are even greater for the school and social domains. For the both autism and SM group, speaking in all domains is further reduced, particularly in social settings. The apparent severity of SM may be increased by the presence of autism, possibly due to increased levels of anxiety associated with autism, or potentially, language/communication impairments related to SM and autism may be additive. Age may also be a factor, as described above. Autistic children may also be less inclined to contribute to conversation topics that do not interest them. The SMQ has been shown to be a reliable instrument for identifying SM (Oerbeck et al., 2020); however, it does not assess anxiety levels, nor does it investigate from where difficulties with communication may stem. The questions focus on when and with whom the child speaks and how much it interferes with their lives.

When considering parental views, the range of SMQ scores was reduced across all domains for groups of children with suspected autism only or neither diagnosis. This suggests that there may still be children who have SM or are both autistic with SM but are yet to be diagnosed, emphasizing the importance of taking parental views into account during diagnosis.

### 4.3. Life Interference

Scoring the life interference questions of the SMQ shows interesting differences in how concerned children and their parents are by non-speaking behavior in relation to diagnosis or parental views on their children’s diagnostic status. Parents report that autism-only children are only a little more concerned about their lack of speech than children with neither diagnosis, whilst for children with SM only, there is nearly always life interference, except within their own families, where their scores still show that they are concerned on some occasions (autism only vs. SM only, *p* = 0.001). Life interference is even greater for those who have both autism and SM diagnoses, but not significantly so (SM only vs. both, *p* = 0.08). The exploratory use of a scoring system for life interference, as performed here, shows a strong correlation with the total SMQ score. This score has not been validated, and future validation work is needed to determine its usefulness.

### 4.4. Freezing Behavior and Factors Affecting Difficulties with Speaking

The small number of responses to the child survey prevents conclusions from being drawn; however, the data do provide insight into the experiences of the children who responded. The children with neither diagnosis nor a suspected diagnosis rarely, if ever, experienced difficulties speaking or freezing-like symptoms. One child, with a notably lower SMQ score than the others in this group, reported extensive freezing. This suggests possible borderline SM, potentially inherited from a parent who reported difficulty speaking themselves on some occasions. The low SMQ score could be reflective of the child’s age (5 years old), but this would not necessarily explain the greater freezing behavior, which tends to be relatively stable over time (Held et al., 2022).

In contrast to the children with neither diagnosis, the children and young people with a diagnosis of SM reported having trouble speaking and regularly experienced freezing of the throat and other body parts. The children with neither diagnosis, but their parent suspected that they had SM, had similar experiences to those with a diagnosis of SM. The low SMQ scores for these children support the parents’ suspicions of SM being present. Vogel et al., using network analysis of symptoms and, separately, a parent survey, also found that immobility of the body in the form of freezing accompanied mutism in children and adolescents with SM (Vogel et al., 2022b, 2024).

The children with a dual diagnosis of both autism and SM, or a suspected dual diagnosis, some of whom were home schooled, all had low SMQ scores and experienced problems with their voices not working and throat and body freezing to some degree. Catatonia is well documented to occur in some autistic children (Moore et al., 2022), which could account for the freezing reported. Equally, it may have been caused by their SM, or there may have been an additive effect from both their SM and autism. Children with both autism and SM experienced throat or body freezing more frequently than children with SM only; however, the absence of responses from autism-only children regarding freezing behavior means it is not possible to gain any insight into whether SM and/or autism is causing their freezing symptoms. The question remains as to whether the mechanism causing catatonia in autism is, in fact, the same as that causing freezing in SM, and whether either is a result of the Defense Cascade. Since neither catatonia nor the Defense Cascade as conceptual frameworks are tested as explanations of freezing behavior in this study, more research is needed to answer these questions.

The children who described having problems speaking, with or without freezing, most of whom had a diagnosis of SM or their parents suspected they had SM, said that their problems were exacerbated by pressure being put on them to speak (impatience from the person expecting them to speak, repetition of questions, requests to hurry up). They reported having had the problem either all their lives, or it started at an age when they would probably have been entering a new environment, such as starting nursery or transitioning to infant, junior, or secondary school. A change in environment, particularly to a more challenging one, is known to be a potential trigger factor for SM (Johnson & Wintgens, 2016).

Other factors affecting difficulties with speaking were varied. Responses from the older children (fear of what the other person would think of what they said, fear of making mistakes, fear of a croaky/frozen voice) reflected their greater awareness of how they are perceived by others (which increases with age). They may also reflect a degree of social anxiety, which can develop if SM is left untreated (Kamani & Monga, 2020). Throat tightening, as described by some children, should be considered as a possible physical factor potentially preventing speech brought on by stress or anxiety. Allowance should be made for this when attempting to elicit speech from children with SM, particularly in cases where a low SMQ score is found or there is an additional autism diagnosis. The effect on any physical task should also be considered, such as the ability to hold a pen and write when frozen or undertake physical activities, since freezing of multiple body parts was reported by young people with SM. This underlines the importance of using methods to reduce their overall stress or anxiety as a first step in any treatment plan for SM and on any occasion when dealing with a person with SM. Providing a consistent non-anxiety-provoking environment (including minimizing pressure to speak) is vital before attempting to encourage communication, elicit speech, or perform other physical tasks. This applies particularly to autistic children with SM because they may also be prone to sensory or environmental overwhelm in addition to anxiety in social situations and speech and communication issues from their SM.

### 4.5. Differences Between Boys and Girls

The girls were, on average, older than the boys, and there was a higher proportion of girls with SM or both autism and SM. These differences between the sexes were reflected in the responses on freezing behavior and stress factors, and they prevented any conclusions from being drawn in terms of differences between the biological sexes.

### 4.6. Limitations and Recommendations for Future Research

Whilst over 200 parents completed the parent survey, they were a self-selected group, and many had an interest in SM, leading to a higher proportion of children with SM than would be expected from a survey of the general population. The use of social media and website invitations to recruit participants prevented families who do not use social media or do not have access to the internet from taking part, thereby limiting the representativeness of the study cohort. Sampling from a wider population, more representative of the population as a whole, would likely give greater proportions of neurotypical and autistic individuals (since autism is more prevalent in the general population than SM). Responders were also predominantly from the UK, further limiting our ability to generalize the findings. A high proportion of parents started the survey but stopped after the introductory questions (Figure 1). When they did continue and answered the questions about their children, the survey was well completed.

The number of children completing surveys was small. Eighty-two parents gave permission for their children to complete a survey; however, only 30 children then went on to do so (three were omitted from analysis, as it was not possible to link the child and parent data). Just one child with a diagnosis of autism only responded, and they did not answer any of the questions about freezing behavior. The responders were a self-selected group, which was imbalanced for age and diagnosis by biological sex; however, the SMQ scores of the responders were similar to those who did not respond, suggesting that the responders are a representative sample of the wider study group. The size of the sample was insufficient to allow for statistical analysis of differences between the sexes, whilst accounting for these confounding factors. Recruitment of a larger study group, stratified by biological sex, age, and diagnosis, would allow for a more informed evaluation of differences between the sexes.

The strong associations found between SMQ scores and interference/freezing behaviors observed may partly reflect shared method variance (all parent-/child-report data), and the child-report data, while valuable, are too sparse for robust subgroup comparisons.

Over two-thirds of the respondents to the child survey were aged under 11 years, with no responses from young people aged 13 or 14 years. This may be reflective of the age at which their anxiety is greatest due to greater pressures on them as young teenagers, particularly for those with diagnoses of SM or autism, or that they are not interested in filling in research questionnaires or are less willing to comply with parental requests than younger or older children. Equally, some parents may have changed their minds about asking their children to complete the survey once they had seen it, for fear of upsetting them, or simply could not find the time to do it. Some children abandoned the child survey partway through. This may be indicative of problems that SM or autistic children may have in using forms of non-verbal communication, or they may have been fearful of filling in questionnaires. Other reasons may be that they were too anxious or did not see the point in the questions, for example, if they did not have freezing behaviors or if they were not interested enough to complete the survey. Ways to make surveys more interesting and accessible to children should be explored for use in future research. Questions in the child survey about freezing behavior were designed to be clear about what was meant by freezing, but it is possible that some children, especially the younger ones, may not have fully understood what they were being asked. We cannot exclude the possibility that psychological reasons, possibly related to BI, were interpreted by some children as freezing rather than physical causes (Fox et al., 2021).

Independently validating the diagnostic information given by the parents was beyond the scope of this study, which is a further limitation. Currently, there is no single quantitative diagnostic tool that can fully confirm a diagnosis of autism, as the tools available can lead to misdiagnosis if used alone (Hus & Segal, 2021). To gain an autism diagnosis within the UK, the National Institute for Health and Care Excellence (NICE) guidelines must be followed. These guidelines require the use of multidisciplinary teams, the application of gold standard tools, and adherence to diagnostic criteria (DSM and ICD). Whilst it is unlikely that parents would declare a clinical diagnosis that does not exist, it is possible they could have misinterpreted the question. However, the pattern of SMQ scores for the different groups would suggest that the parents understood that they were being asked for clinical diagnoses. For future research, the use of a diagnostic tool for autism to help validate a declared diagnostic status is recommended, accepting that no single tool can confirm a diagnosis of autism and that misdiagnosis or false positives are possible, particularly in anxious populations.

Failure to record the biological sex of the child in the parent survey is a further limitation, as it did not allow us to compare parent responses for boys and girls, examine the sex distribution in the two survey cohorts, or identify any differences in the sex declared by parents and their children.

Data was not collected on diagnoses other than SM and autism, so it was not possible to assess whether the children had other co-existing conditions, such as other anxiety disorders, learning or developmental issues, or sensory processing issues, which may have influenced the results, The children’s responses to what made their difficulty with speaking worse may give some clues as to the underlying reasons for their SM, which are likely to be complex and vary from individual to individual. Fear of speech, a predisposition to anxiety, overreactive freeze response, sensory overwhelm, problems with social communication, learning difficulties, BI, co-existing anxiety disorders, or autism could all be contributory factors. More research is needed in this important area.

The associations and trends observed in this study require further evaluation in a larger sample. This would also give a better assessment of the extent of freezing in neurotypical (neither diagnosis) and autistic only children compared with those with SM only and those with both diagnoses.

Ten of the families who responded to the survey had more than one child with a diagnosis or suspected diagnosis, and not all children had the same diagnosis. Further research is required to examine the familial overlap between SM and autism and to investigate how the likely heritable nature of SM, autism, or both combined may affect conclusions drawn from studies such as this. Data were not requested for children under 5 years or over 16 years, so no inferences could be drawn from the chronological position of the children within the family.

## 5. Conclusions

In this study, a quarter of children with SM were also autistic, suggesting a possible relationship between SM and autism in some individuals. Most children who responded to the small-scale qualitative child survey with a diagnosis of SM only or both autism and SM (or suspected diagnoses) reported some level of throat and body freezing, whereas those with neither diagnosis and no parental suspicions of a diagnosis mostly did not. SMQ scores and life interference scores derived from the parent questionnaire varied with diagnosis/suspected diagnosis; children with both autism and SM diagnoses had the lowest average SMQ score (indicating a greater severity of symptoms) and the highest life interference score (indicating greater interference in their lives), followed by children with a diagnosis of SM only. Children in the autism-only group scored more highly than those with SM only on the SMQ but less than those with neither diagnosis. Interference scores correlated negatively with SMQ scores. Further research is needed to assess the validity and usefulness of scoring interference on the SMQ. On average, children with autism only spoke less than children with neither diagnosis but were not concerned by their lack of speech. Further research involving a larger representative sample of children is needed to confirm these trends.

The most common reasons given by the children that increased their difficulty in speaking were pressure being put on them to talk, worries about how they would be perceived, and fear of making mistakes. Physical freezing of both the throat and other parts of the body may be important factors in preventing speech and movement in children with SM, but these observations are preliminary, as they are based on a very small, self-selected sample, and further research is needed before conclusions can be drawn. Autism should also be considered a possibility and investigated when children present with SM symptoms, particularly for those with very low scores on the SMQ, recognizing that a formal diagnosis requires a comprehensive assessment. Due to the heterogeneous nature of SM, overlapping symptoms with autism and high levels of co-existing disorders, such as SAD, it is advisable to undertake a multidisciplinary assessment for SM. A full profile of needs and co-existing conditions can then be identified so that support can be individualized as early as possible in a child’s life. Failure to do this may result in further problems and mental health disorders developing, which could greatly impact the progress and wellbeing of the child. These problems potentially last into adulthood, leading to reduced life chances and the ability to work and function within society.

## Figures and Tables

**Table 1:** Responders’ country of residence.

Country	N (%)
United Kingdom	140 (93%)
United States of America	3 (2%)
New Zealand	2 (1%)
Ireland	1 (<1%)
France	1 (<1%)
Germany	1 (<1%)
Greece	1 (<1%)
United Arab Emirates	1 (<1%)
**Total**	150 (100%)

Data are numbers and percentages (%).

**Table 2:** Number of children in the family.

Number of Children	N (%)
1	69 (47%)
2	58 (40%)
3	17 (12%)
4	1 (<1%)
5+	1 (<1%)
**Total number of responses**	146 (100%)
**Total number of children (excluding families with 5+ children)**	240

Data are numbers and percentages (%).

**Table 3:** Diagnosis by age (years).

Diagnosis	Under 5	5–7	8–11	12–16	All Children
Neither diagnosis	5 (83%)	34 (50%)	41 (57%)	51 (60%)	131 (57%)
Autism only	0 (0%)	3 (4%)	7 (10%)	10 (12%)	20 (9%)
SM only	1 (17%)	30 (43%)	19 (27%)	11 (13%)	61 (26%)
Both autism and SM	0 (0%)	2 (3%)	4 (6%)	13 (15%)	19 (8%)
**Total autism**	0	5	11	23	39
**Total SM**	1	32	23	24	80
**Total children**	6	69	71	85	231
**Percentage of children with SM in each age band who also have Autism**	0%	6%	17%	54%	24%

Data are numbers and percentages (%). SM = selective mutism.

**Table 4:** Agreement between diagnosis and parental view.

	Clinical Diagnosis				
Parental View of the Child’s Diagnosis	Neither Diagnosis	Autism Only	SM Only	Both Autism and SM	Total
Neither diagnosis	81 (35%)	1 (<1%)	1 (<1%)	0 (0%)	83 (36%)
Autism only	15 (6%)	15 ^#^ (6%)	1 (<1%)	0 (0%)	31 (13%)
SM only	26 (11%)	2 (1%)	47 ^+^ (20%)	0 (0%)	75 (32%)
Both autism and SM	9 (4%)	2 (1%)	12 (5%)	19 * (8%)	42 (18%)
**Total**	131 (57%)	20 (9%)	61 (26%)	19 (8%)	231 (100%)

Data are numbers and percentages (%). SM = selective mutism. Lighter shading indicates where the parent considers the child to be underdiagnosed, and darker shading indicates where the parent considers the child to have an incorrect diagnosis. ^#^ No parental response, agreement assumed (n = 3); ^+^ no parental response, agreement assumed (n = 16); * no parental response, agreement assumed (n = 19).

**Table 5:** Diagnostic status and SMQ scores of the responders and non-responders to the child survey.

	Diagnosis				
Response Status	Neither Diagnosis	Autism Only	SM Only	Both Autism and SM	Total
**Responded to the child survey**	10	1	13	1	25
SMQ total score	2.3	2.8	0.9	0.2 *	1.1
(median, IQR)	(1.0–2.7)		(0.8–1.4)		(0.9–2.1)
**Did not respond to the child survey**	101	15	42	13	171
SMQ total score	2.4	1.4	0.8	0.4 (0.4–0.8)	1.7
(median, IQR)	(1.6–2.9)	(1.1–2.4)	(0.5–1.2)		(0.8–2.5)
**All children**	111	16	55	14	196
SMQ total score	2.4	1.7	0.9	0.4	1.5
(median, IQR)	(1.5–2.9)	(1.2–2.4)	(0.6–1.2)	(0.3–0.8)	(0.8–2.5)

Data are the number, median, and interquartile range (IQR). SMQ = Selective Mutism Questionnaire. * Score from 1 child only; two children in this group were home schooled, so scores were not given for them for the school domain questions. Both children scored 6/33 for the 11 questions in the other domains. Home schooled children were excluded from all analyses of SMQ scores.

**Table 6:** Child survey responses of boys and girls.

Characteristic	Boys (n = 14)	Girls (n = 13)	Total (n = 27)
**Age (years)**	7 (5–9)	10 (6–15)	8 (5–11)
**Clinical diagnosis**			
Neither diagnosis	7/14 (50%)	3/13 (23%)	10/27 (37%)
Autism only	1/14 (7%)	0	1/27 (4%)
SM only	5/14 (36%)	8/13 (62%)	13/27 (48%)
Both autism and SM	1/14 (7%)	2/13 (15%)	3/27 (11%)
**Parental view of diagnosis**			
Neither diagnosis, but suspects SM	2/14 (14%)	1/13 (8%)	3/27 (11%)
SM but also suspects autism	1/14 (7%)	2/13 (15%)	3/27 (11%)
**SMQ**			
SMQ total score ^a^	1.5 (1.1–2.6)	0.8 (0.5–1.3)	1.1 (0.9–2.1)
Interference score ^a^	1.3 (0.7–2.3)	2.4 (1.8–2.8)	2 (0.5–2.5)
**Symptoms occurring at least some of the time**			
Voice will not work	9/13 (69%)	12/13 (92%)	21/26 (81%)
Throat feels tight	4/12 (33%)	9/12 (75%)	13/24 (54%)
Body frozen	5/12 (42%)	9/12 (75%)	14/24 (58%)
**Number of body parts affected**	0.5 (0–1.5)	2 (1.5–4)	1.5 (0–3)
**Body parts affected**			
Face	4/12 (33%)	5/12 (42%)	9/24 (38%)
Neck	3/12 (25%)	5/12 (42%)	8/24 (33%)
Hand	2/12 (17%)	5/12 (42%)	7/24 (29%)
Shoulder	2/12 (17%)	4/12 (33%)	6/24 (25%)
Leg	1/12 (8%)	5/12 (42%)	6/24 (25%)
Arm	1/12 (8%)	4/12 (33%)	5/24 (21%)
Stomach	1/12 (8%)	2/12 (17%)	3/24 (13%)
Chest/heart	0	2/12 (17%)	2/24 (8%)
Mouth	0	1/12 (8%)	1/24 (4%)
**Worried about talking**	9/12 (75%)	11/11 (100%)	20/23 (87%)
**Uncomfortable when expected to speak**	4/12 (33%)	8/11 (73%)	12/23 (52%)

Data are the number (n/N) and percentage (%) or the median and interquartile range (IQR). SM = selective mutism, SMQ = Selective Mutism Questionnaire. ^a^ Children who are home schooled are omitted.

## Data Availability

The datasets presented in this article are not readily available because of time limitations for the parent survey and due to privacy reasons for the child survey. Requests to access the datasets should be directed to shirley@selectivemutism.org.uk.

## Supplementary Materials

The following supporting information can be downloaded at https://www.mdpi.com/article/10.3390/bs16010152/s1, Table S1: SMQ scores by diagnosis; Table S2: ‘Interference/bothersome’ scores by diagnosis, Supplement S1: Study questionnaires.

## Abbreviations

The following abbreviations are used in this manuscript:

CEN	Clinical Excellence Network
CNTNAP-2	Contactin-associated protein-like 2
DD	Dissociative disorder
DSM	Diagnosis and Statistical Manual
ICD	International Classification of Diseases
IQR	Interquartile range
NASEN	National Association for Special Educational Needs
NDD	Neurodevelopmental disorder
SAD	Social anxiety disorder
SD	Standard deviation
SM	Selective mutism
SMiRA	Selective Mutism Information and Research Association
SMQ	Selective Mutism Questionnaire
UK	United Kingdom
