# Supplementary material for: Physical Freezing in Children and Adolescents with Selective Mutism
Source: Behav Sci (Basel). 2026 Jan 21;16(1):152. doi: 10.3390/bs16010152 (PMC12837469; doi:10.3390/bs16010152)
Supplement: Supplementary file 1 [file behavsci-16-00152-s001.zip › behavsci-3835125-supplementary.pdf]

Physical Freezing in Children and Adolescents with Selective Mutism

Supplement

Table S1 SMQ scores by diagnosis

Diagnosis	Mean (SD)	Median (IQR)	Min	Max	N
**None**					
Family domain	2.44 (0.71)	2.83 (2.17 – 3)	0	3	99
School domain	2.14 (0.95)	2.5 (1.5 – 3)	0	3	99
Social domain	1.78 (0.99)	1.8 (1 – 3)	0	3	99
Total score	2.14 (0.82)	2.35 (1.53 – 2.88)	0	3	99
**Autism only**					
Family domain	2.11 (1.02)	2.42 (1.33 – 3)	0	3	14
School domain	1.69 (0.84)	1.75 (1.17 – 2.33)	0	3	14
Social domain	1.46 (1.06)	1.4 (0.4 – 2.4)	0	3	14
Total score	1.77 (0.92)	1.12 (2.12 – 2.47)	0	3	14
**Selective mutism only**					
Family domain	1.61 (0.78)	1.5 (1.17 – 2.17)	0	3	50
School domain	0.67 (0.64)	0.5 (0.17 – 1)	0	3	50
Social domain	0.46 (0.57)	0.2 (0 – 0.8)	0	2.2	50
Total score	0.94 (0.55)	0.88 (0.59 – 1.35)	0	2.65	50
**Both**					
Family domain	1.01 (0.59)	1.08 (0.58 – 1.33)	0	2	12
School domain	0.29 (0.33)	0.17 (0 – 0.58)	0	.83	12
Social domain	0.17 (0.32)	0 (0 – 0.2)	0	1	12
Total score	0.51 (0.35)	0.41 (0.24 – 0.82)	0	1.06	12
**All children with a diagnosis of autism or selective mutism**					
Family domain	1.61 (0.86)	1.5 (1.08 – 2.17)	0	3	76
School domain	0.80 (0.78)	0.67 (0.17 – 1.17)	0	3	76
Social domain	0.59 (0.78)	0.2 (0 – 1)	0	3	76
Total score	1.02 (0.72)	0.88 (0.5 – 1.38)	0	3	76

Table S2 ‘Interference/bothersome’ scores by diagnosis

Diagnosis	Mean (SD)	Median (IQR)	Min	Max	N
None	0.80 (0.95)	0.33 (0 – 1.67)	0	3	99
Autism only	0.80 (0.69)	0.92 (0 – 1.5)	0	1.83	14
Selective mutism only	2.18 (0.59)	2.17 (1.83 – 2.67)	0	3	50
Both	2.44 (0.36)	2.5 (2.25 – 2.67)	0	3	12
All children with a diagnosis of autism or selective mutism	1.96 (0.81)	2 (1.5 – 2.5)	0	3	76

Supplement S1 Study questionnaires

Info sheet

Hello & welcome

The Selective Mutism Information and Research Association (SMIRA) became a UK Registered Charity in 1992, having been set up initially to support families with children with/who have selective mutism. This work has since been extended to provide information to health and education professionals involved in the upbringing of such children and young people. Selective Mutism (SM) is an anxiety-based condition that usually commences in early childhood. Those with SM speak fluently in some situations but remain consistently silent or are unable to speak freely in others. SM has been the topic of discussion and research for decades, but there are still things that we do not know, and this impedes the support that can be offered to individuals who experience it. In order to carry on our valuable work, it is now important that we conduct research to fill in some of the gaps in this knowledge.

Why is this research being done?
People with SM often report that their throats feel blocked or tight when expected to speak. Some describe feeling frozen, with muscles in their bodies becoming stiff, limiting their movement. The extent to which SM involves physical freezing is not known. This research is designed to assess the extent to which people with SM and/or Autism or neither condition, may experience freezing behaviour when expected to speak. **It is important that we speak to some children who sometimes find it difficult to speak, and to some who don't usually have those problems in order to find differences that may lead to ways to help.**

Why have I been invited to take part?
You have been asked to take part because **you are a parent/carer of a child or young person who is between the ages of 5 and 16.**

What will taking part involve?
On the following screen you will be asked for your consent to take part in this study. After this you will be asked a series of questions. This should take no more than 10 minutes, **per child,** for you to complete. At the end of that we will request your consent to allow your child/young person to take part in the study. If you consent then we will send you a link to the child/young person survey. We will ask you to guide your child/young person through the various stages to ensure they understand the study and what they are being asked to do at each stage and to ensure that they are happy to answer the questions and do not become distressed. **Please note, results of the individual questionnaires will not be fed back to participants and therefore participants will not receive a diagnosis of SM or autism as part of the study.**

Do I have to take part?
Participation in this study is **voluntary,** there is no payment for taking part and you may withdraw yourself or your child at any stage. **If you or your child do not wish to take part, or change your minds after you have started, just close your browser.**

Data Protection and Transparency
Yours and your child's data will be processed in accordance with the data protection law and will comply with the UK General Data Protection Regulation 2018 (UK GDPR). The data controller for this project will be SMIRA. SMIRA will process your personal data for the purpose of the research outlined above. The legal basis for processing your personal data for research purposes under the data protection law is a 'task in the public interest'.

All information gathered will be kept confidential and only the researchers working on the project will have access to your data. Your name and your child's name will not be mentioned in any published report of the study. Raw data will be kept in a locked cabinet and on a password-protected computer, which will only be accessible to the researchers. In the case of the data being used for academic publication, materials may be kept until ten years have passed from the date of publication.

What are your choices about how the information you provide is used?
You can withdraw your child from the study at any time, without giving a reason. We need to manage your records in specific ways for the research to be reliable.
You can choose to have any data that you provide withdrawn by contacting Researcher 3 (details below) up until one month after supplying it when analysis of the data will have begun, and withdrawal of data is no longer possible.

You can find out more about how we use your information here:

- http://www.selectivemutism.org.uk/privacy-policy/
- https://speechandlanguage.org.uk/privacy-policy/
- by sending an email to research@selectivemutism.org.uk

What are the possible benefits of taking part?
Taking part will potentially inform practice and education for children who have Selective Mutism and/or Autism. This will further lead to a better understanding of the challenges that come with having these two conditions.

Are there any risks?
There are no anticipated risks of taking part, although it is important to consider that **your child taking part will not lead to a diagnosis of either Selective Mutism or Autism.**

What will happen to the results of the research?
The results of this research study will be published as a peer-reviewed research article on an open-access platform.

The results will also be published on the SMIRA social media and web pages.

Ethics
This study has been assessed and approved by the South Birmingham Research Ethics Committee. IRAS Number: 313169. We will adhere to ethical guidelines issued by the British Educational Research Association and the British Psychological Society.

What do I do if I want to know more about the study before I consent to take part?
Contact Researcher 3 (details below) who will be happy to give you more information on the study and answer any questions you may have.

Where can I get more information and advice or support with Selective Mutism?
The SMIRA website www.selectivemutism.org.uk has a large amount of information which can be accessed and downloaded for free. **You can also contact SMIRA directly via the site for specific advice via our website contact page: selectivemutism.org.uk/contact/** .

Thank you very much for taking the time to read this.
Chief Investigator: Dr. Shirley Landrock-White
Email: Shirley@selectivemutism.org.uk
Researcher 2: Victoria Roe
Email: vroe@supanet.com
Researcher 3: Lindsay Lenton
Email: Research@selectivemutism.org.uk

This study is funded by The Communication Consortium Grants Programme (funded by The Rayner's Special Educational Trust) and administered by Speech and Language Uk.

Code Generation

Please generate a code using the last 2 letters of your first name, the month that you were born and the last half of your postcode (tor example if Dave Smith was born in August and lived in the PL49DD area his code would be: VEAUGUST9DD).

Please write this code down as this will be needed for the second survey.

Consent &. SES

Please check each box to confirm your agreement with the statements. Press next in order to proc e ed . If you do not consent, please exit this survey by exiting your browser.

If you have any further questions, please contact researcher 3 below.

	I confirm that
I confirm that I have read the information on the previous screen. I have had the opportunity to consider the information, ask questions and, if asked, have had these answered satisfactorily.	0

	I confirm that
I understand that my participation is voluntary and that I am free to withdraw at any time without giving a reason and without my rights being affected.	0
If I do decide to withdraw my information, I understand that I can only do that up to 30 days after giving it	0
I understand that I will be fully protected in accordance with the **UK** General Data Protection Regulation 2018 (UK GDPR) Act of 2018 and in compliance with the British Educational Research Association and British Psychological Society Ethical Guidelines and that my data will be kept confidential and anonymous until they are securely destroyed.	0
I understand that my name and any personal information collected about me will be anonymised in any report based on this study.	0
I agree that any of the data I provide may be used in the researcher's report and for possible publication in academ ic journals.	0
In case the data are used for publication , I understand that they will be kept for ten years after the article has been published and then destroyed	0
I agree to take part in the study	0

Chief Investigator: Dr. Shirley Landrock-White
Email: Shirley@selectivemutism.org.uk
Researcher 2: Victoria Roe
Email: vroe@supanet.com
Researcher 3: Lindsay Lenton
Email: Research@selectivemutism.org.uk

Consider that the ladder that I am showing you below represents the place that people occupy in society.
At the **top of this ladder** are the people who have **more money, more education,** and **jobs with more recognition.**
At the **bottom of the ladder** are the people who have **less money, less education,** and **jobs with less recognition** or are **long-term unemployed.**
The higher you consider yourself on this ladder, the closer you will be to the people who are at the top of the ladder, and the lower, closer you will be to people who find themselves at the bottom.

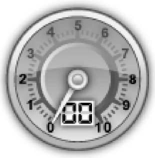

In which country do you currently reside?

Number of Children

Ho w many children between the ages of 5 and 16 do you have?

-
- 2
- 3
- 4
- 5 or more children

If you have more than 4 children, please lea ve your email address and exit the survey. The research team will contact you personally.

Age of Child 1

Thinking about your child/ren, please tell us how old they are.

If you have more than 1 child, you may find it easier, for the purpose of this survey to consider them in age order.

How old is Child l?

How old is Child 2?

How old is child 3?

How old is child 4?

One Child

These questions ask about your child's/young person's current diagnosis.

Does your child have a diagnosis of Autism?

0 Yes

Q No

Does your child have a diagnosis of Selective Mutism?

0 Yes

Q No

Do you think that they **might** have **Selective Mutlsm or Autism?**

Q Yes, Selective Mutism

0 Yes, Autism

0 Yes, both

Q No.

Two Children

These questions ask about your children's current diagnosis. Please think about each child in turn.

Does **child 1** have a diagnosis of **Autism?**

0 Yes

Q No

Does **child 1** have a diagnosis of **Selective Mutism?**

0 Yes

Q No

Do you think that **child 1** might have **Selective Mutlsm or Autism?**

Q Yes, Selective Mutism

0 Yes, Autism

0 Yes, both

Q No.

Does child 2 have a diagnosis of **Autism?**

0 Yes

Q No

Does child 2 have a diagnosis of **Selective Mutism?**

Q Yes

Q No

Do you think that child 2 might have **Selective Mutism or Autism?**

Q Yes, Selective Mutism

0 Yes, Autism

0 Yes, both

Q No.

Three Children

These questions ask about your children's current diagnoses. Please think about each child in turn.

Does child 1 have a diagnosis of **Autism?**

0 Yes

Q No

Does child 1 have a diagnosis of **Selective Mutism?**

Q Yes

Q No

Do you think that **child 1** might have **Selective Mutism or Autism** ?

0 Yes, Selective Mutism

0 Yes, Autism

Q Yes, both

Q No.

Does **child 2** have a diagnosis of **Autism?**

0 Yes

Q No

Does **child 2** have a diagnosis of **Selective Mutism?**

0 Yes

Q No

Do you think that **child 2** might have **Selective Mutism or Autism?**

Q Yes, Selective Mutism

Q Yes, Autism

0 Yes, both

Q No.

Does **child 3** have a diagnosis of **Autism?**

Q Yes

Q No

Does **child 3** have a diagnosis of **Selective Mutism?**

0 Yes

Q No

Do you think that **child 3** might have **Selective Mutism or Autism?**

Q Yes, Selective Mutism

0 Yes, Autism

0 Yes. both

Q No.

Four Children

These questions ask about your children's current diagnoses.

Does **child 1** have a diagnosis of **Autism ?**

0 Yes

Q No

Does **child 1** have a diagnosis of **Selective Mutism?**

0 Yes

Q No

Do you think that **child 1** might have **Selective Mutlsm or Autism?**

Q Yes, Selective Mutism

0 Yes. Autism

0 Yes, both

Q No.

Does **child 2** have a diagnosis of **Autism?**

- Yes
- No

Does **child 2** have a diagnosis of **Selective Mutism?**

- Yes
- No

Do you think that **child 2** might have **Selective Mutism or Autism?**

- Yes, Selective Mutism
- Yes, Autism
- Yes, both
- No.

Does **child 3** have a diagnosis of **Autism?**

- Yes
- No

Does **child 3** have a diagnosis of **Selective Mutism?**

- Yes
- No

Do you think that **child 3** might have **Selective Mutism or Autism?**

- Yes, Selective Mutism
- Yes, Autism

0 Yes, both

Q No.

Does **child 4** have a diagnosis ot **Autism?**

0 Yes

Q No

Does **child 4** have a diagnosis ot **Selective Mutlsm?**

0 Yes

Q No

Do you think that **child 4** might have **Selective Mutism or Autism?**

0 Yes, Selective Mutism

0 Yes, Autism

0 Yes, both

Q No.

SMQ 1 child

This questionnaire asks you about your child/ children's speaking behaviours in different environments. You will be asked to complete it tor each ot your children. In reference to your **first or only child**:

Please consider **your first/only** child's beha vior in the last two weeks and rate how frequently each statement is true tor **your first/only** child.

At School:

	Always	Often	Seldom	Never	My child is home schooled
I. When appropriate, my child talks to most peers at school.	0	0	0	0	0

	Always	Often	Seldom	Never	My child is home schooled
2. When appropriate, my child talks to selected peers (his/her friends) at school.	0	0	0	0	0
3. When my child is asked a question by his/her teacher, s/he answers.	0	0	0	0	0
4. When appropriate, my child asks his or her teacher questions.	0	0	0	0	0
5. When appropriate, my child speaks to most teachers or staff at school.	0	0	0	0	0
6. When appropriate, my child speaks in groups or in front of the class.	0	0	0	0	0

Please consider your **first/only chl d's** behavior in the last two weeks and rate how frequently each statement is true for **your first/only child**.

At home or with family:

	Always	Often	Seldom	Never
7. When appropriate, my child talks to family members living at home when other people are present.	0	0	0	0
8. When appropriate, my child talks to family members while in unfamiliar places.	0	0	0	0
9. When appropriate, my child talks to family members that don't live with him/her (e.g., grandparent, cousin).	0	0	0	0
10. When app ropriate, my child talks on the phone to his/her parents and siblings.	0	0	0	0
II. When appropriate, my child speaks with family friends who are well-known to him/her.	0	0	0	0

	Always	Often	Seldom	Never
12. My child speaks to at least one familiar visitor to the home. Eg. babysitter, family friend.	0	0	0	0

Please consider **your first/only child's** behavior in the last two weeks and rate how frequently each statement is true for your **first/only child .**

In social situations (outside of school).

	Always	Often	Seldom	Never
13. When appropriate, my child speaks with other children who s/he doesn't know.	0	0	0	0
14. When appropriate, my child speaks with family friends who s/he doesn't know.	0	0	0	0
15. When appropriate, my child speaks with his or her doctor and/or dentist.	0	0	0	0
16. When appropriate, my child speaks to shop assistants and/or waiters.	0	0	0	0
17. When appropriate, my child talks when in clubs, teams, or organized activities outside of school.	0	0	0	0

Thinking about **your first/only child** in general,

	Always	Often	Seldom	Never
18. How much does not talking interfere with school for your child?	0	0	0	0
19. How much does not talking interfere with family relationships?	0	0	0	0
20. How much does not talking interfere in social situations for your child?	0	0	0	0
21. Overall, how much does not talking interfere with life for your child?	0	0	0	0

	Always	Often	Seldom	Never
22. Overall, how much does not talking bother your child?	0	0	0	0
23. Overall, how much does your child's not talking bother you?	0	0	0	0

SMQ Child 2

Please think about **Child 2's** Speaking Behaviours whilst answering these questions.

Please consider **child's 2** behavior in the last two weeks and rate how frequently each statement is true **for your second** child.

At School:

	Always	Often	Seldom	Never	My child is homesch oled
I. When appropriate, my child talks to most peers at school.	0	0	0	0	0
2. When appropriate, my child talks to selected peers (his/her friends) at school.	0	0	0	0	0
3. When my child is asked a question by his/her teacher, s/he answers.	0	0	0	0	0
4. When appropriate, my child asks his or her teacher questions.	0	0	0	0	0
5. When appropriate, my child speaks to most teachers or staff at school.	0	0	0	0	0
6. When appropriate, my child speaks in groups or in front of the class.	0	0	0	0	0

Please consider **child's 2** behavior in the last two weeks and rate how frequently each statement is true **for your second** child.

At home or with family:

	Always	Often	Seldom	Never
7. When appropriate , my child talks to family members living at home when other people ore present.	0	0	0	0
8. When appropriate, my child talks to family members while in unfamiliar places.	0	0	0	0
9. When appropriate, my child talks to family members that don't live with him/her (e.g., grandparent, cousin).	0	0	0	0
10. When appropriate, my child talks on the phone to his/her parents and siblings.	0	0	0	0
ll. When approp ria te, my child speaks with family friends who ore well-known to him/her.	0	0	0	0
12. My child speaks to at least one babysitter.	0	0	0	0

Please consider **chi d's 2** behavior in the last two weeks and rate how frequently each statement is true **for your second** child.

In social situations (outside of school).

	Always	Often	Seldom	Never
13. When appropriate, my child speaks with other children who s/he doesn't know.	0	0	0	0
14. When appropriate , my child speaks with family friends who s/he doesn't know.	0	0	0	0
15. When app ropriate , my child speaks with his or her doctor and/or dentist.	0	0	0	0
16. When approp riate , my child speaks to shop assistants and/or waiters.	0	0	0	0

	Always	Often	Seldom	Never
17. When app ropriate , my child talks when in clubs, team s, or organized activities outside of school.	0	0	0	0

Thinking about **your second child** in general,

	Always	Often	Seldom	Never
18. How much does not talking interfere with school for your child?	0	0	0	0
19. How much does not talking interfere with family relationships?	0	0	0	0
20. How much does not talking interfere in social situations for your child?	0	0	0	0
21. Overall, how much does not talking interfere with life for your child?	0	0	0	0
22. Overall, how much does not talking bother your child?	0	0	0	0
23. Overall, how much does your child's not talking bother you?	0	0	0	0

SMQ Child 3

Please think about **child 3's** speaking behaviours whilst answering these questions.

Please consider **child 3's** behavior in the last two weeks and rate how frequently each statement is true for **your third child** .

At School:

	Always	Often	Seldom	Never	My child is homeschooled
I. When appropriate, my child talks to most peers at school.	0	0	0	0	0

	Always	Often	Seldom	Never	My child is homeschooled
2. When appropriate, my child talks to selected peers (his/her friends) at school.	0	0	0	0	0
3. When my child is asked a question by his/her teacher, s/he answers.	0	0	0	0	0
4. When appropriate, my child asks his or her teacher questions.	0	0	0	0	0
5. When appropriate, my child speaks to most teachers or staff at school.	0	0	0	0	0
6. When appropriate , my child speaks in groups or in front of the class.	0	0	0	0	0

Please consider **child 3's** behavior in the last two weeks and rate how frequently each statement is true for **your third child** .

At home or with family:

	Always	Often	Seldom	Never
7. When appropriate, my child talks to family members living at home when other people are present.	0	0	0	0
8. When appropriate, my child talks to family members while in unfamiliar places.	0	0	0	0
9. When appropriate, my child talks to family members that don't live with him/her (e.g., grandparent, cousin).	0	0	0	0
10. When appropriate , my child talks on the phone to his/her parents ond siblings.	0	0	0	0
ll. When appropriate, my child speaks with family friends who are well-known to him/her.	0	0	0	0
12. My child speaks to at least one babysitter.	0	0	0	0

Please consider **child 3's** behavior in the last two weeks and rate how frequently each statement is true for **your third child** .

In social situations (outside of school).

	Always	Often	Seldom	Never
13. When appropriate, my child speaks with other children who s/he doesn't know.	0	0	0	0
14. When appropriate, my child speaks with family friends who s/he doesn't know.	0	0	0	0
15. When app ropriate , my child speaks with his or her doctor and/or dentist.	0	0	0	0
16. When appropriate, my child speaks to shop assistants and/or waiters.	0	0	0	0
17. When appropriate, my child talks when in clubs, teams, or organized activities outside of school.	0	0	0	0

Thinking about **your third child** in general,

	Always	Often	Seldom	Never
18. How much does not talking interfere with school for your child?	0	0	0	0
19. How much does not talking interfere with family relationships?	0	0	0	0
20. How much does not talking interfere in social situations for your child?	0	0	0	0
21. Overall, how much does not talking interfere with life for your child?	0	0	0	0
22. Overall, how much does not talking bother your child?	0	0	0	0
23. Overall, how much does your child's not talking bother you?	0	0	0	0

SMQ Child 4

Please think about child's 4's speaking behaviours whilst answering these questions.

Please consider child 4's behavior in the last two weeks and rate how frequently each statement is true for your fourth child.

At School:

	Always	Often	Seldom	Never	My child is homesch oled
I. When appropriate. my child talks to most peers at school.	0	0	0	0	0
2. When appropriate, my child talks to selected peers (his/her friends) at school.	0	0	0	0	0
3. When my child is asked a question by his/her teacher, s/he answers.	0	0	0	0	0
4. When appropriate, my child asks his or her teacher questions.	0	0	0	0	0
5. When appropriate, my child speaks to most teachers or staff at school.	0	0	0	0	0
6. When appropriate, my child speaks in groups or in front of the class.	0	0	0	0	0

Please consider child 4's behavior in the last two weeks and rate how frequently each statement is true for your fourth child.

At home or with family:

	Always	Often	Seldom	Never
7. When appropriate, my child talks to family members living at home when other people are present.	0	0	0	0
8. When appropriate. my child talks to family members while in unfamiliar places.	0	0	0	0
9. When appropriate, my child talks to family members that don't live with him/her (e.g., grandparent. cousin).	0	0	0	0

	Always	Often	Seldom	Never
10. When app ropriate , my child talks on the phone to his/her parents and siblings.	0	0	0	0
11. When appropriate, my child speaks with fam ily friends who are well-known to him/her.	0	0	0	0
12. My child speaks to at least one babysitter/.	0	0	0	0

Please consider **child 4's** behavior in the last two weeks and rate how frequently each statement is true for **your fourth child**.

In social situations (outside of school).

	Always	Often	Seldom	Never
13. When appropriate, my child speaks with other children who s/he doesn't know.	0	0	0	0
14. When appropriate, my child speaks with family friends who s/he doesn't know.	0	0	0	0
15. When appropriate, my child speaks with his or her doctor and/or dentist.	0	0	0	0
16. When appropriate, my child speaks to shop assistants and/or waiters.	0	0	0	0
17. When appropriate, my child talks when in clubs, teams, or organized activities outside of school.	0	0	0	0

Thinking about your **fourth child** in general,

	Always	Often	Seldom	Never
18. How much does not talking interfere with school for your child?	0	0	0	0
19. How much does not talking interfere with family relationships?	0	0	0	0

	Always	Often	Seldom	Never
20. How much does not talking interfere in social situations for your child?	0	0	0	0
21. Overall, how much does not talking interfere with life for your child?	0	0	0	0
22. Overall, how much does not talking bother your child?	0	0	0	0
23. Overall, how much does your child's not talking bother you?	0	0	0	0

Family History

These questions ask about some of your family history.

Please **read the statement and If it applies to your family,** type the persons/ peoples relationship to your child/children in the text bo xes.

There is a further text box belo w if you should need it.

Extended family in this instance **means** ***biologically related parents, aunts/uncles and grandparents to your children.***

	Person I: Relationship to child	Person 2: Relationship to child	Person 3: Relationship child	Person 4:Relationship to child
Have any members of your extended family had Selective Mutism in the past?				
Do any members of your extended family have a current diagnosis of Selective Mutism?				
Do any members of your extended family have a diagnosis of Autism?				
Do any members of your extended family have difficulty talking in some situations (now or in the past) but do not have a diagnosis of Selective Mutism or Autism?				

	Person l: Relat ion ship to child	Person 2: Relationship to child	Person 3: Relationship child	Person 4:Relationship to child
Do any members of your fam ily have, or have had, diagnoses of other mental health or Neurodiverse conditions?				

What mental health condition did your child's $ {q: / / QID89 / ChoiceTextEntryValue/ 8/1} have?

- ☐ Anxiety
- ☐ Depression
- ☐ Po st natal depression
- ☐ Other

What mental health condition/s was it? Please list them all if more than one.

What mental health condition did your child's $ {q: / / QID89 / ChoiceTextEntryValue/ 8/ 2} have?

- ☐ Anxiety
- ☐ Depression
- ☐ Post natal depression
- ☐ Other

What mental health condition/s was it? Please list them all if more than one.

What mental health condition did your child's$ {q://QID89/ChoiceTextEntryValue/8/3} have?

0 Anxiety

D Depression

D Post natal depression

D Other

What mental health condition/s was it? Please list them all if more than one.

What mental health condition did your child's $ { q: / / QID89 / ChoiceTextEntryValue/ 8/ 6} have?

D Anxiety

D Depression

D Post natal depression

0 Other

What mental health condition/s was it? Please list them all if more than one.

If there are any more people within your extended family, that you haven't already told us about, please do so below.

Note: Please include relationship to child and what mental health condition was diagnosed.

Child Survey Permissions

Would you be willing to sit with your child/ children whilst they fill in a short survey with a few questions on speaking behaviours?

0 Yes

Q No

If you consent for your child or children to take part in a similar survey please check each box to confirm your agreement with the statements. Press next in order to proceed . If you do not consent, please exit this survey by exiting your browser .

I confirm that I will support my child in taking part in a similar survey	0
I understand that my child's participation is voluntary and that they are free to withdraw at any time without giving a reason and without their rights being affected.	0
If I do decide to withdraw my child at the end of the study, I understand that the information that they have given can only be removed up to one month after it has been given.	0
I understand that my child will be fully protected in accordance with the UK General Data Protection Regulation 2018 (UK GDPR) Act of 2018 and in compliance with the British Educational Research Association and British Psycho logical Society Ethical Guidelines and that my data will be kept confidential and anonymous until they are securely destroyed.	0
I understand that my child's name and any personal information collected about them will be anonymised in any report based on this study .	0
I agree that any of the data that my child provides may be used in the researcher's report and for possible publication in academic journals.	0
In case the data ore used for publication, I understand that they will be kept for ten years after the article has been published and then destroyed.	0

I agree for my child to take part in the above study

0

In order for your children to take part you will be sent a further link/s. We have separate surveys for children who are 5-10 years and young people aged 11-16.

Please note: You will need to open the relevant link separately for each child.

Please now leave your email address so that the research team can send you the link to the child/young person questionnaire.

Alternatively please email the research team with your interest in the follow up survey's on research@selectivemutism.org

Debrief

Please download a hard copy of this debrief here: Smira debrief study 2 v3 and store it on your computer. This way, if you require the contact details at a later date you will have access to this.

Thank you for helping out with this study. The main aim of this research is to investigate the physical nature of selective mutism. In this study, we were interested in how Selective Mutism and Autism may coexist in some people and may, or may not, be interrelated. We hope you have enjoyed taking part and found the study interesting. We will be happy to answer any further questions you may have.

Remember, all data is completely confidential and will be reported as group statistics only. Your name and your child's name will not be associated with your data and neither of you will be named in any publications or presentations of the research. You have the right to withdraw from the study at any time without penalty. If you decide to withdraw at a later date, please Email Researcher 3 citing the unique ID code that you generated at the start of the study as this will allow us to identify and withdraw your anonymised data.

If this study has raised any personal issues which you would like to have information or talk to someone about then the following websites may be of help to you:

The National Autistic Society: www.autism.org.uk
The NHS pages for Selective Mutism: www.nhs.uk/mental-health/conditions/selective-mutism and for Autism: www.NHS.uk/conditions/autism

Whilst SMIRA does not give psychological or psychotherapy support directly, **it does give advice on how to find this kind of support see this link** : Info: Where to Get Help with Selective Mutism - SMIRA

You can also contact one of our SMIRA team members directly for specific advice via our website contact page: selectivemutism.org.uk/contacU.

For more immediate relief this website contains some well-being activities: https://www.childrenssociety.org.uk/information/young-people/well-being/activities

Thank you again for taking part If you have any concerns about this research, please contact researcher three in the first instance. If your concern is not dealt with adequately then please contact the Chief Investigator.

Chief Investigator: Dr Shirley Landrock-White
Email: Shirley@selectivemutism.org.uk

Researcher 2: Victoria Roe
Email: vroe@supanet.com

Researcher 3: Lindsay Lenton
Email: Research@selectivemutism.org.uk

Powered by Qualtrics

SMiRA
Challenging Selective Mutism

Registered Charity No. 1022673
VAT No. 221 0698 36

www.selectivemutism.org.uk

Information Sheet for Children Aged 5-10

We would like to ask you to help us with our research study

Please read this or ask someone to read it to you.

You can talk to your mum, dad, or carer if there is anything you don’t understand.

Take time to decide if you want to join in

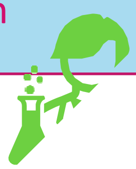

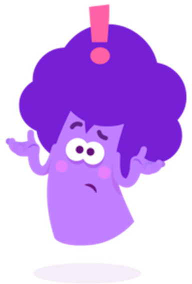

Why are we doing this study?

We are doing this study so that we can learn about children who find it hard to speak sometimes and then we can help them.

Why have I been asked to help?

We are asking you to help us because we need to ask as many children and young people as we can.

Do I have to help?

No, you do not have to help with this study. It is up to you. Your Mum, Dad or your carers can help you decide. You can stop at any time.

What might be hard about helping with the study?

If you help us, you will need to answer some questions on the computer or a phone which might take a little bit of time to do. You won't have to talk to us at all, just tick some boxes or write a few words or get someone to help you write them if you need to.

What are the good things about taking part?

Some children like taking part in studies because they know it will help other children in the future.

What will happen when the study stops?

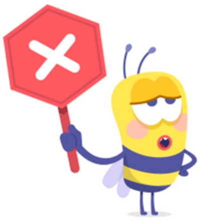

Research studies can take quite a long time to complete, but if your Mum, Dad or carer gives us an email address, we will write to them and let you know what we find out when we finish the study.

What will happen to all the information about me?

All the information you tell us will be kept private. We won't tell anyone your name or what you told us about yourself, and we will make it so no one can find them out. We will look at all the information you and other children have given us and write about it to help more children.

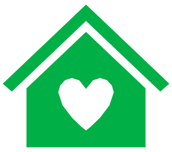

What if there is a problem?

We will try and help with any problems you may have. If you get upset your Mum, Dad or carer will be there to help you. They can talk to SMIRA for you and help you answer the questions if you need them to!

SMiRA

c alle t setecttve Mutt s,',\.

Please remember that you will need to open the link to the survey separately for each of your children that are aged 5- 10 years old.

Parent/carer please enter the code that you generated at the beginning of your survey. As a reminder this was made up of the last 2 letters of your first nam e, the month that you were born and the last half of your postcode (for example if Dave Smith was born in August and lived in the PL49DD area his code would be: VEAUGUST9DD).

Please open the inform at ion sheet below and read this with **your** child.

Sm ir□ information sheet for 5 IDs study 2 vl

Press here to go to the next page

Have you downloaded the information sheet and had a discussion with your child about this survey?

O I have downloaded and shared the information sheet with my child and I have ensured their understanding and comfort with the task

Press here to go bock

Press here to go to the next page

How old are you?

0 5

0 6

0 7

0 8

0 9

0 10

Press here to go back

Press here to go to the next page

Are you a boy or a girl?

0 Boy

0 Girl

Press here to go back

Press here to go to the next page

Do you speak more than one language?

0 No

0 Yes, 2

0 Yes more than 2

Press here to go bock

Press here to go to the next page

The following questions ask you about what happens when you talk.

Press here to go bock

Press here to go to the next page

When I try to talk my voice won't make a sound. won't work. something stops me from sp eaking.

Always	Often	Sometimes	Rarely	Never
0	0	0	0	0

Press here to go bock

Press here to go to the next page

When I try to talk my throat feels different as if it is tig ht, frozen , blocked, or as if there is a lump in it, so it is difficult to get words out

Always	Often	Sometimes	Rarely	Never
0	0	0	0	0

Press here to go bock **Press here to go to the next page**

Other parts of my body feel frozen or stiff if I try to move them

Always	Often	Sometimes	Rarely	Never
0	0	0	0	0

Press here to go bock **Press here to go to the next page**

Which parts?

Face	Neck	Shoulders	Arms	Hands	Legs	Other
☐	☐	☐	☐	☐	☐	☐

Press here to go bock **Press here to go to the next page**

Do you have any other uncomfortable feel ings in your body when you are expected to speak?

0 Yes

0 No

Press here to go bock

Press here to go to the next page

Tell us about these uncomfortable feelings.

____

____

Press here to go bock

Press here to go to the next page

I feel worried when people want me to talk?

Always	Often	Sometimes	Rarely	Never
0	0	0	0	0

Press here to go bock

Press here to go to the next page

How long (in years) has this been a problem for you?

____

____

Press here to go bock

Press here to go to the next page

When people want you to talk is there anything you do to help yourself to feel a bit less worried? If so. what?

Press here to go back

Press here to go to the next page

When people want you to talk does anything make you feel even more worried? If so. what?

Press here to go back

Press here to go to the next page

Thank you so much for answering all of these questions for us. If you are worried please speak to your parent or carer and enjoy watching this video.

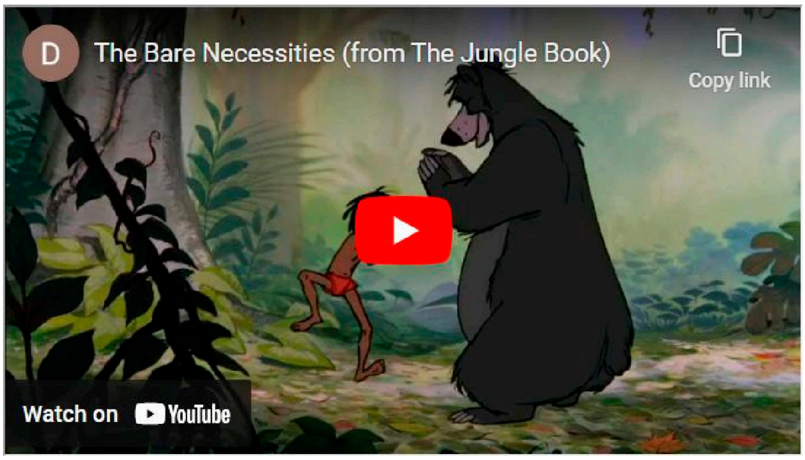

Press here to go back

Press here to go to the next page

SMiRA

Challenging Selective Mutism

we thank you for your time spent taking this survey.
Your response has been recorded.

11 to 16 Questionnaire

SMiRA
Challenging Selective Mutism

Please remember that you will need to open the link to the survey separately for each of your children that are aged 11-16 years.

Parent/carer please enter the code that you generated at the beginning of your survey. As a reminder this was made up of the last 2 letters of your first name, the month that you were born and the last half of your postcode (for example if Dave Smith was born in August and lived in the PL49DD area his code would be: VEAUGUST9DD).

Which one of your children will be responding to this survey?

Press here to go to the next page

Please now allow your child to respond to the rest of this survey

Press here to go back

Press here to go to the next page

Hello &. Welcome,

This research aims to find out more about the relationship between Autism and Selective Mutism. The results will help other people to understand the conditions and support people who have them more effectively.

What is Selective Mutism?

Some people find it very difficult or impossible to speak in some situations or to some people. This is not by choice; their bodies just prevent them from speaking. You can find out lots more about it and how to get advice, help and support with Selective Mutism on the SMIRA website www.selectivemutism.org.uk

Why is this research being done?

Some people find that their bodies physically freeze up in some situations or with some people , so we want to find out how often this happens, and whether it happens more often for people who have Selective Mutism, and/or Autism or neither condition. We also want to find out how this freezing may affect them.

What will taking part involve?

If you are willing to help with the research, we will ask you to complete the consent form and then complete an online survey that consists of [number of] questions.

What will happen at the end of the study?

After the study has concluded you will be given some more information on what the study is about, along with details of relevant organisations that you can contact for more information and support if you feel that this is needed.

What if I am uncomfortable with this?

Your help with the research is your choice, so you may stop taking part at any time. If you do not wish to take part or you wish to stop, you can do so by simply exiting your browser.

What will happen to the answers to my questionnaire?

The answers from your questionnaire will be added to those from others and the results analysed. Later, they will be published through SMIRA and articles in journals, so that other people can use the information to better understand and help young people with Selective Mutism.

Will any readers be able to identify me?

No real names will be used and all responses will be kept securely. Your name will not be attached to your questionnaire and will not be published.

What do I do if I want to know more about the study before I consent to take part?

You can ask your parent/carer to contact the Research team for more information or to ask questions about the study on your behalf.

Who is the researcher?

Selective Mutism Information and Research Association (SMIRA). www.selectivemutism.org.uk

Press here to go back

Press here to go to the next page

This exploratory research study aims to find out more about the physical symptoms of Selective Mutism which may affect the ability to speak in some situations. The results will help people to understand Selective Mutism and how to overcome it.

If you have read the Inform ation Leaflet and are willing to help with the rese arch , we ask you to sign this consent form. Your parent/ carer com pleted a separate consent form. If you would like more information about the study before signing the consent form please ask your parent/ carer to contact the Research team on your behalf.

This study is being done as part of a project funded by The Communication Trust through the Rayner's Special Education Trust. Please note that there should be no risk to you if you choose to take part. All the answers given on the questionnaire are valued. will be kept confidential and will only be used for the purposes of research.

Please check each box to confirm your agreement with the statements. Press next in order to proceed. If you do not consent. please exit this survey by exiting your browser.

I understand that:

	I con firm tha t
It is my choice to take part in this research study and I may stop at anytime.	0
No one will be able to identify me in anything that is writt en about this research.	0

Press here to go back

Press here to go to the next page

How old are you?

0 11

0 12

0 13

0 14

0 15

0 16

Press here to go back

Press here to go to the next page

What was your sex registered at birth?

Male	Female
0	0

Press here to go back

Press here to go to the next page

Do you speak more than one language?

No, only one	Yes, two	Yes, 3+
0	0	0

Press here to go back

Press here to go to the next page

The following questions ask you about your speaking behaviour. Please answer them as honestly as you can.

Press here to go back | **Press here to go to the next page**

I find it physically difficult to speak.

Always	Often	Sometimes	Rarely	Never
0	0	0	0	0

Press here to go back | **Press here to go to the next page**

When I try to talk my throat feels different, as if it is tight, frozen, blocked, or as if there is a lump in it, so it is difficult to get words out

Always	Often	Sometimes	Rarely	Never
0	0	0	0	0

Press here to go back | **Press here to go to the next page**

If there's a chance I might be expected to speak other parts of my body can feel physically frozen or stiff if I try to move them

Always	Often	Sometimes	Rarely	Never.
0	0	0	0	0

Press here to go back | **Press here to go to the next page**

Which parts?

Face	Neck	Shoulders	Arms	Hands	Legs	Other
☐	☐	☐	☐	☐	☐	☐

Press here to go back

Press here to go to the next page

Do you have any other uncomfortable feelings in your body when you are expected to speak?

0 Yes

0 No

Press here to go back

Press here to go to the next page

Tell us about these uncomfortable feelings.

____

____

Press here to go back

Press here to go to the next page

I feel anxious when I am expected to speak in some situations?

Always	Often	Sometimes	Rarely	Never
0	0	0	0	0

Press here to go back

Press here to go to the next page

How long (in years) has this been a problem for you?

Press here to go back

Press here to go to the next page

When people want you to talk is there anything you do to help yourself to feel a bit less worried? If so, what?

Press here to go back

Press here to go to the next page

When people want you to talk does anything make you feel even more worried ? If so, what?

Press here to go back

Press here to go to the next page

SMiRA
Challenging Selective Mutism

Thank you for helping out with this study. The main aim of this research is to investigate the physical nature of selective mutism. We hope you have enjoyed taking part and found the study interesting. We will be happy to answer any further questions you may have.

Remember, all data is completely confidential and will be reported as group statistics only. Your name will not be associated with your data and you will not be named in any publications or presentations of the research. You have the right to withdraw from the study at any time without penalty. **!.f_y:ou decide to withdraw at a later date..P-lease ask your P-arent/carer to email Researcher 3 citing the uni ue ID code** that they generated at the start of the study as this will allow us to identity and withdraw your anonymised data.

If this study has raised any personal issues which you would like to have information or talk to someone about then the following websites may be of help to you:

SMIRA: www.selectivemutism.org.uk

The National Autistic Society_:_ www.autism.org.uk

The NHS P-ages for:

Selective Mutism: www.nhs.uk/ mental-health/conditions/ selective-mutism
Autism: www.NHS.uk/ conditions/ autism.

For more immediate relief this website contains some well-being activities:
https: / / www.childrenssociety.org.uk/ information/ young-people/ well- being/ activities

Thank you again for taking part

Press here to go back

Press here to go to the next page

SMiRA

Challenging Selective Mutism

We thank you for your time spent taking this survey.
Your response has been recorded.
